# Stand-alone Transcriptional Immune Response Prediction in Primary Triple-Negative Breast Cancer

**DOI:** 10.1158/2767-9764.CRC-25-0453

**Published:** 2025-12-15

**Authors:** Suze Roostee, Fredrika Killander, Hani Saghir, Deborah F. Nacer, Jari Häkkinen, Iñaki Sasiain, Srinivas Veerla, Johan Vallon-Christersson, Niklas Loman, Mattias Ohlsson, Johan Staaf

**Affiliations:** 1 Division of Translational Cancer Research, Department of Laboratory Medicine, Lund University, Lund, Sweden.; 2 SUND DataLab, Center for Health Data Science, Department of Public Health, Faculty of Health and Medical Sciences, Copenhagen University, Copenhagen, Denmark.; 3 Division of Oncology, Department of Clinical Sciences Lund, Lund University, Lund, Sweden.; 4 Computational Science for Health and Environment, Centre for Environmental and Climate Science, Lund University, Lund, Sweden.

## Abstract

Triple-negative breast cancer (TNBC) accounts for 10% to 20% of primary breast cancers and often has early relapses and aggressive progression. An activated tumor immune response can be prognostic in patients with treatment-naïve and chemotherapy-treated TNBC and may be assessed using gene expression data. We derived a stand-alone predictor for a proposed immunomodulatory transcriptional TNBC subtype in a training cohort of 235 patients with primary disease based on random forest modeling of RNA sequencing data. Validation in independent TNBC cohorts totaling more than 1,200 patients demonstrated that the classifier recapitulates the immunomodulatory mRNA subtype classification, is associated with elevated immune expression and diversity of T-cell receptor genes, is associated with response to neoadjuvant chemotherapy, and can separate patients into subgroups with better or worse prognosis after adjuvant chemotherapy. The availability of stand-alone classifiers for mRNA-based prediction may further enhance RNA sequencing’s usability in a more routine clinical context and for translational endpoints in clinical trials.

**Significance:**

Tumor immune response has prognostic and treatment predictive value in TNBC and can be estimated by, e.g., mRNA profiling. Translating this association into classifications for single patients requires stand-alone predictors. We have developed one such mRNA classifier that could be applied in future clinical contexts and clinical trials.

## Introduction

Triple-negative breast cancer (TNBC) is a clinical subgroup of breast cancer defined by negativity for the estrogen receptor (ER), progesterone receptor, and *ERBB2/HER2* gene amplification. TNBC constitutes 10% to 20% of patients with primary disease and has historically been associated with a poorer prognosis and earlier relapses compared with other breast cancer subgroups (1, 2). TNBC tumors display great molecular heterogeneity with seemingly different biological pathways underlying the disease (3). Consequently, several subtyping approaches aiming to further delineate TNBC into molecular subtypes of clinical relevance beyond the conventional clinical markers have been reported (4).

Based on analyses of gene expression data, different subtyping schemes have been presented (5–8). Although the number of mRNA subtypes differs between studies, a commonly identified transcriptional feature is the high expression of genes associated with immune response in a subgroup of tumors, exemplified by the immunomodulatory (IM) subtype identified by Lehmann and colleagues (5). The recapitulation of the transcriptional IM subtype in several TNBC studies (4) aligns with findings that TNBC tumors have a higher degree of tumor-infiltrating lymphocytes (TIL) compared with other clinical breast cancer subgroups (9). Elevated TIL levels, as well as mRNA expression of genes associated with immune response, have been shown to be prognostic in both treatment-naïve and chemotherapy-treated patients in multiple studies (10–12). In addition, several gene signatures have been reported focusing on the prediction of pathologic complete response (pCR) in patients with neoadjuvant chemotherapy (NAC)–treated TNBC, many of which include an immune component (13–19). Although the IM subtype has been shown to reflect immune infiltration, it has not yet been proposed as a distinct prognostic signature of its own in TNBC (5, 20). Given the clinical introduction of combined NAC and immune checkpoint therapy (ICT) based on successful clinical trials in TNBC (21, 22), the interest in characterization of the tumor immune microenvironment (TIME) and in immune response as a measurable biomarker in TNBC is increasing, especially considering the lack of robust treatment-predictive markers in the preoperative setting.

A tumor immune response can be estimated in multiple ways, e.g., by TILs scored by pathologists on histologic slides or from the expression of immune response–associated genes in bulk tumor tissue. Irrespectively, a measure of immune response is typically not a bimodal distribution of values from distinct “immune warm” and “immune cold” tumors but rather a continuous distribution of estimates. The latter makes tumor classification attempts inherently dependent on arbitrary cutoffs (often relative) and thus often also on cohort composition. In fact, both the biological composition (e.g., the number of immune “warm”/“cold” tumors and the level of infiltration guided by tissue sampling) and the association with patient outcomes (e.g., whether cohorts are population-representative or not) are influential factors in cohort composition. For gene expression data, arbitrary cutoffs and cohort differences between training and test sets may lead to inconsistencies and suboptimal classification when predictors are applied to independent cohorts, as previously illustrated for nearest centroid (NC)–based classifiers like PAM50 (23).

In this study, we aimed to derive a stand-alone predictor for immune status prediction in TNBC based on gene expression data obtained from RNA sequencing (RNA-seq), with a particular focus on optimized patient prognostication in the training procedure. Although immune status can be estimated or predicted in several ways using gene expression data and training label assignment, we chose to base our predictor development on the NC-based IM subtype originally proposed in 2011 by Lehmann and colleagues (5) and later complemented by an online tool for subtype classification (TNBCtype; ref. 20). Importantly, the current online tool for the Lehmann subtypes does not provide an IM subtype class directly; instead, it returns a correlation value to the IM centroid for each tumor in the submitted cohort (20). This means that results will be relative to the cohort submitted for analysis and that a threshold must be applied to call a subtype for a tumor. In this context, a stand-alone predictor would mitigate these limitations and allow individual samples to be independently and consistently predicted. Validation analyses of the derived stand-alone IM predictor in more than 1200 TNBC cases demonstrate that it can recapitulate a Lehmann IM subtype classification, it is associated with immune expression and with response to NAC treatment, and it can separate patients with TNBC into subgroups with better or worse prognosis after adjuvant chemotherapy.

## Materials and Methods

### Ethics approval and consent to participate

Patients were enrolled in the Sweden Cancerome Analysis Network – Breast (SCAN-B) study (ClinicalTrials.gov ID: NCT02306096; refs. 24, 25), approved by the Regional Ethical Review Board in Lund, Sweden, or the Swedish Ethical Review Authority (registration numbers 2009/658, 2010/383, 2012/58, 2013/459, 2014/521, 2015/277, 2016/541, 2016/742, 2016/944, 2018/267, 2019-01252, and 2024-02040-02). Inclusion and exclusion criteria for SCAN-B patients are outlined in NCT02306096. All patients provided written informed consent prior to enrollment, including for publishing information about sex and age. All analyses were performed in accordance with patient consent and ethical regulations and decisions. This study conformed to the principles of the Helsinki Declaration.

### SCAN-B patient cohorts

Based on our recently reported Swedish population–representative TNBC cohort (26) of patients enrolled in the SCAN-B study, we assembled 235 cases with complete RNA-seq data and extensive treatment and clinical follow-up data. The main chemotherapy regimen for the training cohort was FEC based [combination of 5-fluorouracil, epirubicin (E), and cyclophosphamide (C)] ± a taxane in 96% of cases (26). Patients in this cohort were enrolled from 2010 to early 2015 in the Skåne healthcare region in South Sweden. This patient cohort was used as a training cohort, and so it is referred to as SCAN-B_training hereon (Table 1). PD-L1 combined positive scores (PD-L1 CPS), estimated based on IHC using the 22C3 antibody, were obtained from the study by Sigurjonsdottir and colleagues (27) and whole-slide hematoxylin and eosin TIL estimates from the study by Aine and colleagues (28). CPS were categorized into three groups as CPS 0 (CPS <1), CPS 1 (CPS 1–9), and CPS 2 (CPS ≥10; ref. 27). Matched tissue microarray (TMA)–based digital cell counts calculated with the TMArQ software of six immune cell type markers CD3 (pan-lymphocyte marker), CD20 (B-cell marker), CD4 (T-helper cell marker), CD8 (cytotoxic T-cell marker), FOXP3 (naturally occurring regulatory T-cell marker), and CD68 (macrophage marker) were obtained from Roostee and colleagues (29). Cell counts represent the average count per marker across TMA cores for a sample.

**Table 1.** Clinicopathologic and molecular characteristics of included TNBC cohorts.

Dataset	SCAN-B_training (2010–2015)	SCAN-B_validation (2015–2018)	FUSCC validation	TCGA validation	SCAN-B validationNAC^a^	GSE163882 Chen and colleagues^b^	GSE164458 Metzger-Filho and colleagues
Cohort usage	Training	Validation	Validation	Validation	Validation	Validation	Validation
Original number of patients with TNBC	235	145	362	86	118	89	482
Number of non-TNBC patients	0	0	0	0	0	128	0
Patients with TNBC after ER filtering by TNBCtype used in main analyses	232	142	295	82	116	NA	NA
Patients classified as having metastatic disease after study enrollment	6	0	NA	NA	0	NA	NA
Chemotherapy	​	​	​	​	​	​	​
Adjuvant	174^c^	72	278	22	0	NA	NA
Neoadjuvant	16	34	NA	NA	116	217	482
None	36	36	7	NA	0	0	0
Median patient age (range) in years	62 (26–91)	65 (30–95)	53 (25–81)	55 (35–90)	55 (30–80)	54 (23–82)	NA
Tumor grade	​	​	​	​	​	​	​
I	0	0	0	NA	NA	15	NA
II	25	26	55 + 24^d^	NA	NA	73	NA
III	204	101	189	NA	NA	128	NA
Median tumor size (SD) in mm	20.5 (15.8)	16 (14.2)	25 (12)	NA	NA	NA	NA
Lymph node status	​	​	​	​	​	​	​
Negative	149	96	173	47	NA	NA	288
Positive	83	37	120	27	NA	NA	194
Consensus IM class	​	​	​	​	​	​	​
IM negative	152 (66%)	95 (67%)	182 (62%)	51 (62%)	69 (59%)	172 (79%)	230 (48%)
IM positive	80 (34%)	47 (33%)	113 (38%)	31 (38%)	47 (41%)	45 (21%)	252 (52%)

Abbreviation: NA, data not available.

a Includes an overlap of patients with NAC in the SCAN-B validation cohort enrolled from 2015 to 2018.

b Clinical data summarized for all patients in GSE163882.

c Of the 174 patients, 147 were deemed eligible for survival analysis using IDFS as endpoint according to ref. 26.

d Annotated as grade II to III.

To provide an independent, nonoverlapping, SCAN-B RNA-seq validation cohort, we extracted data from 145 patients with TNBC diagnosed from 2015 to early 2018 in the Skåne healthcare region from the study by Staaf and colleagues (30). An extensive clinical review was performed for these patients to obtain updated treatment and follow-up information. This patient cohort is hereon referred to as SCAN-B_validation. The main chemotherapy regimen given to patients included in this cohort was a combination of sequential anthracycline- and taxane-based regimens, usually EC, followed by a taxane (docetaxel or paclitaxel).

To investigate the treatment-predictive performance of the developed RNA-seq classifier for response to NAC, we assembled a cohort of 118 SCAN-B patients treated preoperatively with standard-of-care chemotherapy during 2013 to 2021 (97% of patients diagnosed after 2015), with available RNA-seq data of a diagnostic pre-treatment biopsy specimen preserved in RNAlater (Invitrogen). This cohort is referred to as SCAN-B_validationNAC. Thirty-three tumors in the SCAN-B_validationNAC cohort overlapped with the SCAN-B_validation cohort. For patients treated with NAC (mainly EC + taxane), pCR was defined as eradication of viable invasive tumor cells in both the breast and lymph nodes (when lymph nodes are assessed, ypT0/is ypN0; ref. 31) based on data from clinical charts; otherwise, the patient was classified as having residual disease (RD). The SCAN-B_validationNAC cohort was used exclusively to assess treatment-predictive performance because of short follow-up time for the majority of included patients. Among the patients with RD in the SCAN-B_validationNAC cohort, 36 patients had available RNA-seq also from posttreatment fresh surgical resection tumor tissue, forming 36 pairs of pretreatment and posttreatment RNA-seq profiles. Clinicopathologic characteristics and molecular classifications for the different SCAN-B cohorts are shown in Table 1, with patient-specific details provided in Supplementary Table S1. TIL estimates, *in situ* IHC immune cell marker counts, and PD-L1 CPS scores were only available for the SCAN-B_training cohort.

### Gene expression analyses

Gene expression profiling of tumors in the SCAN-B_training, SCAN-B_validation, and SCAN-B_validationNAC cohorts was performed using RNA-seq as previously described and summarized as fragments per kilobase per million mapped fragment (FPKM) values (30). Gene expression data and PAM50 subtypes for tumors in the SCAN-B_training and SCAN-B_validation cohorts were obtained from the online repository associated with the study by Staaf and colleagues (30). Remaining RNA-seq data for SCAN-B tumors were generated and processed using the same analysis setup as in Staaf and colleagues (30). FPKM values for the SCAN-B_validationNAC patients are provided as Supplementary Table S2. FPKM values from the SCAN-B_training and SCAN-B_validationNAC (*n* = 36 patients with RD) cohorts were used to impute fractions of 22 immune cell types through RNA-seq deconvolution with CIBERSORTx (32). Each cohort was analyzed separately online with their webtool and the parameters LM22 signature matrix file, batch correction enabled (B-mode), quantile normalization disabled, absolute mode, and 100 permutations. CIBERSORTx *P* values were compared with a threshold of 0.05 as lower *P* values are connected to a more reliable deconvolution process.

### Public adjuvant TNBC gene expression cohorts

To validate findings in external cohorts, we collected two datasets from open-source repositories. Based on the study by Staaf and Aine (33), we gathered FPKM and clinical data (TIL estimates were not available) for 86 TNBCs from The Cancer Genome Atlas (TCGA) consortium. This patient cohort is hereon referred to as TCGA_validation. From the study by Jiang and colleagues (7), performed at the Fudan University Shanghai Cancer Center (FUSCC) in China, we retrieved clinical data, TIL estimates, and matched RNA-seq FASTQ files for 362 patients from the original study and from the Sequence Read Archive (PRJNA486023). FASTQ files were run through our gene expression analysis pipeline described in Staaf and colleagues (30) with minor changes to software tool versions (Trimmomatic version 0.33 and Bowtie version 2.2.9; refs. 34, 35) to create an FPKM expression matrix containing 19,675 genes. This patient cohort is hereon referred to as FUSCC_validation. Clinicopathologic characteristics and molecular classifications for both cohorts are shown in Table 1, with patient-specific details provided in Supplementary Table S1.

### Public NAC TNBC gene expression cohorts

Processed gene expression data and clinical annotations from two reported NAC RNA-seq datasets, GSE163882 (*n* = 217, mRNA expression as FPKM; ref. 36) and GSE164458 (*n* = 482 TNBCs with pretreatment biopsies and mRNA expression as reads per kilobase million; refs. 37, 38), were obtained from Gene Expression Omnibus (GEO; RRID:SCR_005012). Treatment response data were available as pCR or RD. GSE163882 comprised 89 TNBCs, 27 ER-negative and HER2-positive tumors, 36 ER-positive and HER2-positive tumors, and 65 ER-positive and HER2-negative tumors. GSE164458 comprised patients with TNBC randomized to three separate treatment arms, labeled as A (carboplatin plus the PARP inhibitor veliparib concurrent with paclitaxel), B (paclitaxel followed by doxorubicin/cyclophosphamide plus carboplatin), and C (paclitaxel followed by doxorubicin/cyclophosphamide). Analysis of treatment response in GSE164458 was performed for all cases combined and for each treatment arm separately.

### TNBC cell line gene expression data

RNA-seq data for 34 TNBC cell lines were obtained from the study by Jovanović and colleagues (39) as processed FPKM expression values (GEO, series GSE202770).

### Single-cell RNA-seq data from TNBC tumors and normal breast tissue

Processed, compiled, and cell-phenotyped single-cell RNA-seq (scRNA-seq) data in the form of R Seurat data objects for five TNBC cases originally reported by Wu and colleagues (40) were obtained from the study by Pang and colleagues (41). Cell phenotypes included B cells, endothelial cells, epithelial cells, malignant cells, myeloid cells, stromal cells, and T cells. Additional scRNA-seq data from normal breast tissue, together with cell type annotations, were obtained from Reed and colleagues (42) for 803,283 epithelial, stromal, and immune cells as deposited in the CELLxGENE platform (43).

### Immune metagene rank scores and gene set enrichment analysis

For included RNA-seq cohorts, we computed, per cohort and tumor, rank score sums for the immune response metagene proposed by Fredlund and colleagues (44) based on the approach originally described by Nacer and colleagues (45). In this context, the immune response metagene consists of 71 highly, positively inter-correlated genes associated with different immune pathways defined from a gene network analysis. In Nacer and colleagues (45), rank scores were computed individually for each tumor based on FPKM values without any additional normalization or data centering, i.e., they represent individual sample scores. Gene set enrichment analysis was performed on genes with an importance score > 0 in the final random forest model using *enrichR* version 3.2 and the GO_Biological_Process_2023 and KEGG_2021_Human databases. An adjusted *P* value < 0.05 was used as significance threshold.

### TNBCtype IM classification

To assign suitable training labels to samples with a focus on optimized patient prognostication for TNBCtype IM status, we combined survival data and consensus labels from iterated NC classifications in the SCAN-B_training cohort. To classify tumors as TNBCtype IM positive (“immune warm”) or IM negative (“immune cold”), the NC-based TNBCtype webtool was used (20). Briefly, gene expression data for each cohort to be analyzed were uploaded to the webtool for correlation to the original six Lehmann subtypes: basal-like 1, basal-like 2, IM, mesenchymal, mesenchymal stem-like, and luminal androgen receptor. If the webtool returned samples as being estimated as ER positive, these were removed from the cohort (and thus excluded from analysis, see Table 1) and the reduced cohort was uploaded again for correlation with the centroids. To create more stable IM labels for a tumor, gene expression data for a cohort were split into six different folds using the KFold function in *scikit-learn* (shuffle = True and random_state = 42). Every sample in the full training set was left out on one of the six folds, resulting in every sample being present five times. The online tool TNBCtype provided classification on all six folds, with a total of five labels for every sample. If the correlation with the IM centroid for a tumor in a fold analysis was > 0.17, then the sample was considered IM positive, otherwise IM negative. The combination of all labels for a sample determined its final class: if the sample was considered IM positive three or more times, it resulted in a consensus IM-positive label, otherwise in a consensus IM-negative label. The coefficient cutoff was set to 0.17 as this value was the highest correlation cutoff with the highest HR in univariate Cox regression analysis in our training cohort (Supplementary Fig. S1). All data, including training and independent test data, were IM-classified accordingly.

### T-cell receptor and B-cell receptor repertoire analysis

RNA-seq FASTQ files for SCAN-B_validation and FUSCC_validation tumors were processed with MiXCR version 4.5.0 using the *rna-seq* preset and specifying species as *hsa* (46, 47). For the divergence analysis, we used *post-analysis* and *individual* presets, enabling the calculation of CDR3 metrics, Shannon–Wiener diversity measurement, and gene segment usage for all tumors as a single group in the respective cohort.

### Training of a stand-alone classifier for IM status

**Figure 1.**
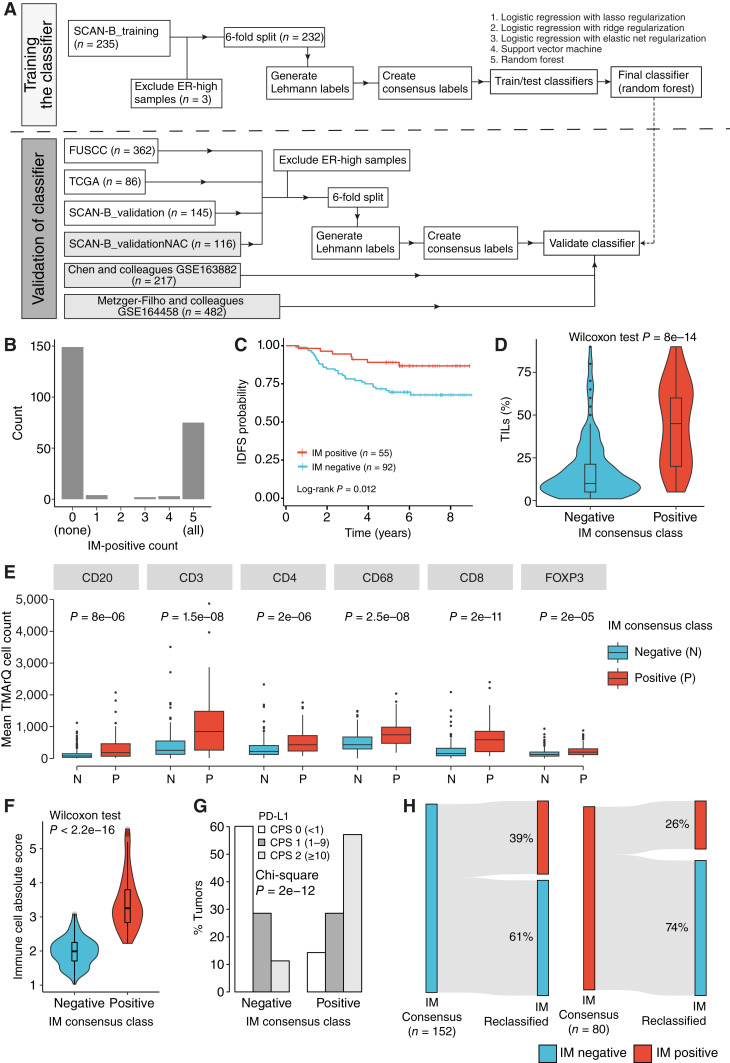
Outline of IM predictor development and TNBCtype IM status in the SCAN-B_training cohort. **A,** Outline of the training and validation schemes for prognosis and treatment prediction. Datasets used for validation of treatment prediction are marked in gray. Dataset sizes correspond to total cohorts. Not all cases have available clinical outcome data or a TNBC phenotype. **B,** Lehmann IM classification count for every sample in the six-fold split on the full training cohort using random subsets. **C,** Kaplan–Meier plot using IDFS as clinical endpoint vs. IM consensus class. **D,** Violin plot of TIL percentage scores (based on pathology evaluation of whole-slide hematoxylin and eosin stains) vs. the IM consensus class groups. **E,** Boxplots of digital immune cell counts from TMArQ analysis of single-plex IHC stainings of immune cell markers on matched TMA cores collected from Roostee and colleagues (29). Cell counts represent the average count of available TMA cores for a sample. Two-sided *P* values calculated using a Wilcoxon test. **F,** Violin plot of total immune cell absolute score estimated based on RNA-seq deconvolution with CIBERSORTx for 22 immune cell types vs. the IM consensus class groups. One IM-negative sample was excluded because of deconvolution. *P* value > 0.05. **G,** Proportions of PD-L1 CPS calculated from IHC staining vs. IM consensus class. **H,** Sankey plot of reclassification results when performing TNBCtype classification using only tumors with a negative IM consensus class (left) or a positive class (right).

The outline for the model development is shown in Fig. 1A. For the initial phase of model training, the SCAN-B_training cohort underwent a sample split of 70% (*n* = 163) for training and 30% for validation (*n* = 69) to facilitate model selection and hyperparameter optimization. Various models were evaluated as potential models, including logistic regression with lasso, ridge, or elastic net regularization, a support vector machine, and a random forest. The feature set was limited to genes present across all internal and external datasets (i.e., SCAN-B_training, SCAN-B_validation, TCGA_validation, and FUSCC_validation). For the training labels, we used Lehmann IM consensus labels as outlined above. To assess whether the number of training samples available was adequate, we measured the model performance with an increasing number of training samples. The training set was sampled four times at sizes 50, 100, 150, and 163 (all) samples, and the performance was averaged while keeping the validation set fixed. This analysis indicated that the number of samples available for training was sufficient (Supplementary Fig. S2A). Subsequently, employing a five-fold cross-validation approach, the random forest model emerged as the optimal choice, demonstrating superior performance and stability (Supplementary Fig. S2B). Following model selection, the random forest model underwent a final training iteration using the full training dataset. Final hyperparameter settings were 50 trees as determined with a grid search, with a maximum depth of six. Model development and final model training were carried out in Python version 3.7.12 using *scikit-learn* version 1.0.2.

### Survival analyses

Survival analyses were performed in R version 4.3.1 using the survival package with invasive disease–free survival (IDFS) as endpoint in SCAN-B cohorts, overall survival (OS) as endpoint in TCGA, and recurrence-free survival as endpoint in FUSCC based on available clinical data. Survival curves were compared using Kaplan–Meier estimates and the log-rank test. Univariate and multivariate Cox regression were performed to estimate HRs using the coxph R function. Covariates adjusted for in multivariate Cox regression included patient age at diagnosis (<50 years or not), Nottingham histologic grade (NHG), and lymph node status (negative/positive).

## Results

### TNBCtype IM status is associated with patient outcomes and immune infiltration but is not an intrinsically stable classification

To analyze the stability of a TNBCtype IM classification in the SCAN-B_training cohort, we first created IM consensus labels using the online TNBCtype tool by a six-fold randomization approach (Fig. 1A). As shown in Fig. 1B, 223 of 232 (96.1%) cases possible to subtype by the online tool had a consistent IM call (all negative or all positive) based on this approach. The resulting consensus labels (IM negative and IM positive) showed a significant association with patient survival in the SCAN-B_training cohort using IDFS as clinical endpoint for patients treated with adjuvant chemotherapy (log-rank test *P* = 0.012; Fig. 1C). Moreover, IM-positive tumors were significantly associated with elevated TIL levels (Fig. 1D), elevated digital *in situ* cell counts of immune cell markers CD3 (pan-lymphocyte marker), CD20 (B-cell marker), CD4 (T-helper cell marker), CD8 (cytotoxic T-cell marker), FOXP3 (naturally occurring regulatory T-cell marker), and CD68 (macrophage marker; Fig. 1E), elevated immune cell presence scores estimated based on RNA-seq deconvolution (Fig. 1F), and PD-L1 CPS based on 22C3 antibody stains (Fig. 1G). The former suggests that gene expression–based IM status can represent a proxy for, e.g., TIL levels in primary TNBC tumor tissue.

Although the randomized consensus label approach demonstrated stability in IM status, it has repeatedly been shown that non-randomized perturbations (e.g., excluding large numbers of samples of a single subtype, followed by reclassification) can drastically change NC subtype calls (23, 48). To demonstrate that this also applies to TNBCtype IM status, we divided the SCAN-B_training cohort into two subsets based on the IM consensus class, one IM-positive cohort (*n* = 80) and one IM-negative cohort (*n* = 152), and reclassified each cohort separately online. As shown in Fig. 1H, reclassification changed a substantial proportion of originally IM-positive and IM-negative tumors to the opposite class. These results highlight that cohort composition has a significant effect on the TNBCtype classification of individual samples. An additional indication of the inherent issues with NC-based subtyping schemes is the similar TNBCtype IM status proportions we observed across independent cohorts used in this study (Table 1) despite them being of different sizes and demographic origins. Taken together, although a TNBCtype IM phenotype seems clearly associated with improved patient outcomes after adjuvant chemotherapy in population-representative primary TNBC, the current subtyping scheme shows limitations in the classification implementation.

### Deriving a stand-alone classifier of IM status based on RNA-seq data

**Figure 2.**
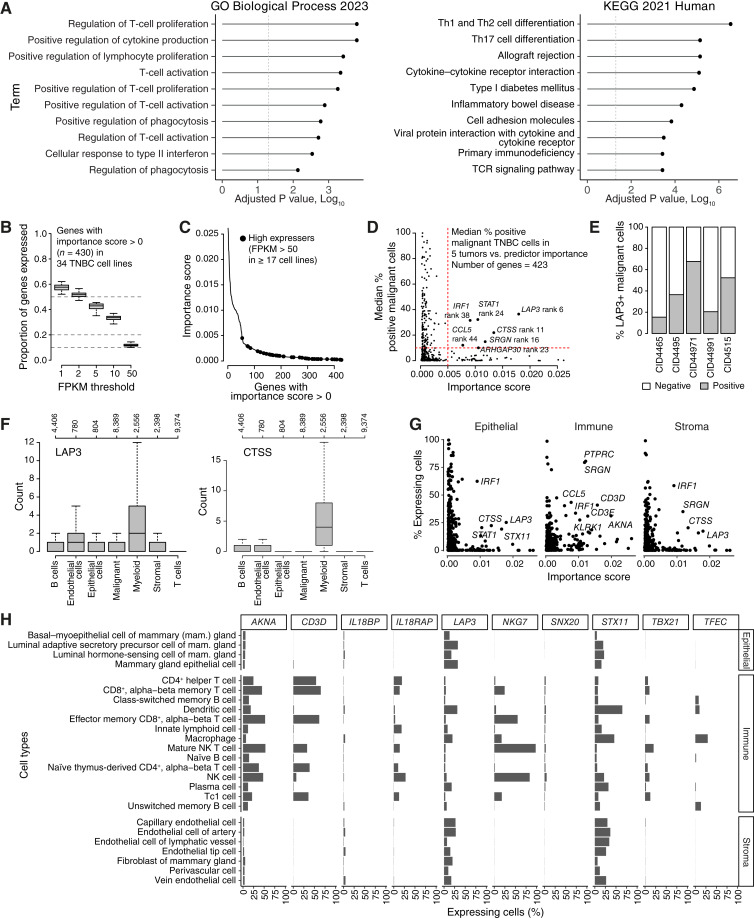
Expression of predictor genes in cell lines and scRNA-seq data. **A,** Gene set enrichment analysis results for 433 genes with predictor importance score > 0 using Gene Ontology (GO) biological processes (left) or Kyoto Encyclopedia of Genes and Genomes (KEGG) terms (right). Dotted vertical line indicates an adjusted *P* value of 0.05. **B,** Proportion of the 430 predictor genes (430 of 433 available for analysis) with a predictor importance score > 0 that are expressed above different FPKM thresholds in 34 TNBC cell lines. At an FPKM threshold of 50, only approximately 12% of the 430 genes have FPKM levels equal to or higher than the cutoff in the cell lines. **C,** Predictor importance score of highly expressed predictor genes in cell lines. The line plot shows the ordered importance score for the 430 predictor genes with scores > 0. Genes with FPKM > 50 in 17 or more of the 34 cell lines are marked by black dots. **D,** Scatter plot of predictor gene importance scores for 423 genes (of the 433 in total) vs. median percent of positive cells phenotyped as malignant from five scRNA-seq TNBC tumors. A positive malignant cell for a gene in a tumor was defined as having a specific gene count > 0. Seven genes with higher importance scores and positive malignant cell percentages are marked, including their importance score rank (rank 1 = highest importance). **E,** Percent of LAP3-positive (LAP3+, *LAP3* count > 0) and -negative cells phenotyped as malignant in five scRNA-seq–profiled TNBC tumors. **F,***LAP3* and *CTSS* scRNA-seq count values vs. cell phenotypes for seven cell types identified in five TNBC tumors. Numbers above boxplots correspond to the number of cells available. **G,** Percentages of several cell types grouped by epithelial/immune/stromal compartments expressing a given gene calculated from an initial pool of 803,283 cells. A cell was counted as expressing a gene whenever its scRNA-seq count was > 0. Almost all (432 of 433) genes with importance score > 0 are displayed. **H,** Similar to (**G**) but displaying percentages by the different cell types and only for the 10 genes with highest importance score for the predictor.

To address the inherent classification issues with NC-based classifiers, we trained a stand-alone classifier (i.e., a classifier that needs a gene expression vector of only one sample) based on IM consensus labels and FPKM data in the SCAN-B_training cohort with subsequent validation in independent TNBC cohorts (Fig. 1A). Although the final random forest classifier is based on 17,529 genes, only 433 genes have an importance score > 0 in the model (Supplementary Table S3). A gene set enrichment analysis of the 433 genes with importance score > 0 revealed, as expected, a strong enrichment for genes associated with immune response including the Kyoto Encyclopedia of Genes and Genomes pathways T-cell receptor (TCR) signaling and Th1, Th2, and Th17 cell differentiation, as well as multiple Gene Ontology Biological Processes related to T cells (adjusted *P* value < 0.05; Fig. 2A). Consistently, several genes among the 10 with highest scores have been previously associated with immune response in cancer such as *CD3D*, *NKG7*, *SNX20*, *IL18RAP*, and *IL18BP* (49–52).

To investigate the putative tumor-intrinsic and -extrinsic expression patterns of the 433 predictor genes with importance score > 0, we first analyzed RNA-seq FPKM data from 34 TNBC cell lines (39), representing a context free of the tumor microenvironment and TIME. In the 34 cell lines, nearly 40% of genes do not seem to be expressed at all whereas slightly more than 10% have an FPKM level >50 (chosen as an arbitrary value of high expression) and might thus be considered higher expressed (Fig. 2B). Notably, the genes with highest expression (FPKM > 50) in at least 50% of cell lines showed typically lower importance scores (Fig. 2C), suggesting that predictor genes with highest importance scores have a mainly tumor-extrinsic expression pattern.

To substantiate this hypothesis, we next analyzed five TNBC tumors profiled by scRNA-seq (*n* = 28,707 cells; ref. 41), acknowledging that the small sample size may influence generalizability of any findings. In this set of five tumors, 29.2% (*n* = 8,389) of all sequenced cells were phenotyped as “malignant.” For malignant cells in each tumor, we computed the percentage of positive cells (gene count >0) and plotted the median of these tumor percentages versus the predictor importance for each of the 423 available genes with predictor importance > 0. As seen in Fig. 2D, only a limited number of genes with higher importance scores seemed to be typically expressed in cells phenotyped as malignant across the five tumors. Although *LAP3* (ranked six in predictor importance) represented the most distinct case in this analysis, it showed a positive expression (count > 0) in approximately 20% to 65% of malignant cells in the individual tumors, suggesting potential tumor heterogeneity (Fig. 2E). Moreover, *LAP3*, as well as *CTSS* (ranked 11 in importance), showed noticeable expression also in nonmalignant cell populations when compared across the five tumors (Fig. 2F). To investigate in which nonmalignant cell types these genes were expressed, we used a reported scRNA-seq dataset including more than 800,000 cells from the immune, stromal, and epithelial compartments of normal breast tissue samples (42). Most genes with importance score > 0 were expressed in multiple cell types (Fig. 2G), and the genes with highest importance scores were more often expressed in immune cells compared with stromal/epithelial cells (Fig. 2H; Supplementary Fig. S3). Together, these results suggest that the developed predictor mainly captures and relies on a tumor-extrinsic gene expression pattern.

### IM predictor classification performance in independent TNBC cohorts

**Figure 3.**
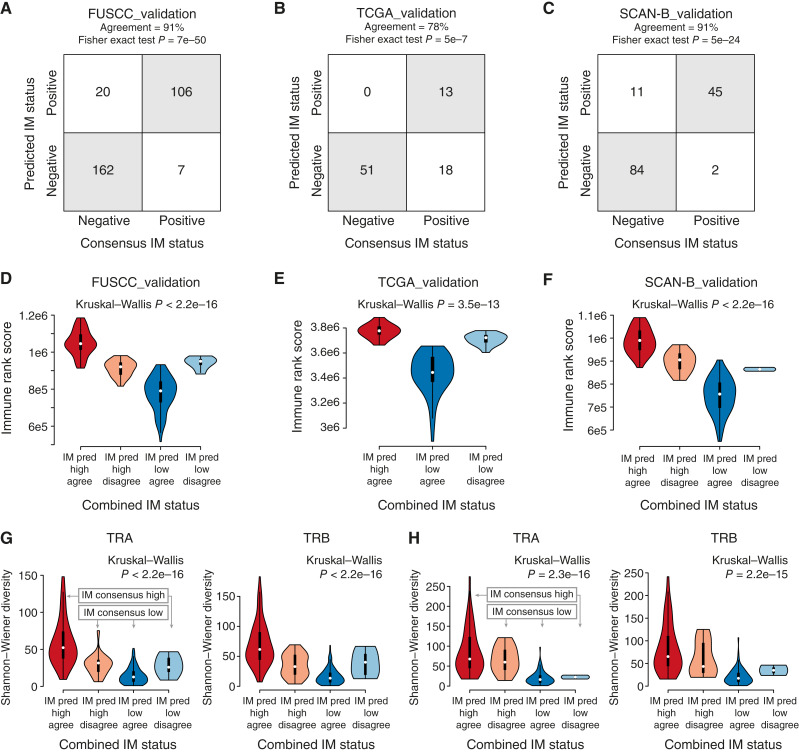
Classification performance and immune gene expression association of the IM classifier in independent TNBC cohorts. **A,** Confusion matrix of IM consensus labels and predicted IM labels in the FUSCC_validation cohort. **B,** Confusion matrix of IM consensus labels and predicted IM labels in the TCGA_validation cohort. **C,** Confusion matrix of IM consensus labels and predicted IM labels in the SCAN-B_validation cohort. **D,** Violin plots of immune response rank score vs. a four-tier label derived from combination of IM consensus and predicted IM labels in the FUSCC_validation cohort. **E,** Same analysis as in (**D**) but in the TCGA_validation cohort. **F,** Same analysis as in (**D**) but in the SCAN-B_validation cohort. **G,** Violin plots of Shannon–Wiener diversity score based on MiXCR analyses for the TCR genes *TRA* and *TRB* vs. a four-tier label derived from combination of IM consensus and predicted IM labels in the FUSCC_validation cohort. **H,** Same as in (**G**) but in the SCAN-B_validation cohort.

To assess the classification performance of the IM predictor, we applied it to FPKM data from three independent TNBC cohorts, FUSCC_validation, TCGA_validation, and SCAN-B_validation, and compared subtype prediction results with corresponding TNBCtype IM consensus labels for each cohort (Fig. 1A). As seen in Fig. 3A–C, the IM predictor showed agreement with the consensus labels in the range of 78% to 91% across the three cohorts. To further analyze the classifier’s prediction performance, we combined predicted IM subtypes with the TNBCtype consensus labels into a four-tier label for which we analyzed rank scores of the Fredlund and colleagues (44) immune response metagene (Fig. 3D–F), which has been shown to strongly correlate with pathology-estimated TIL counts in the SCAN-B_training cohort (29). This analysis demonstrated higher immune rank scores in IM-positive tumors compared with IM-negative cases across cohorts and that discordantly classified samples typically showed intermediate rank scores consistent with a more borderline phenotype. We performed the same comparison with the probability of predicted IM-positive status to further showcase that discordant samples are predominantly borderline cases (Supplementary Fig. S4). Although TIL estimates were not available for the TCGA_validation and SCAN-B_validation cohorts, predicted IM-positive tumors in the FUSCC_validation cohort were significantly associated with higher stromal TIL levels (two-sided Wilcoxon test, *P* = 1e−11), consistent with the RNA-seq–based immune response metagene results and findings in the training cohort. To further analyze the predicted IM subtype association with immune features, we derived Shannon–Wiener diversity indexes for TCR genes (TCR: *TRA*, *TRB*, *TRD*, and *TRG*) and B-cell receptor (BCR) genes (BCR: *IGH*, *IGK*, and *IGL*) based on TCR/BCR repertoire analysis of available bulk tumor RNA-seq data in the SCAN-B_validation and FUSCC_validation cohorts. This analysis demonstrated higher diversity scores for TCR genes (mainly *TRA* and *TRB*; Fig. 3G and H), but not for the BCR genes, in IM-positive tumors (Supplementary Fig. S5A and S5B for all genes).

### Association of the IM predictor with patient outcomes in independent TNBC cohorts

**Figure 4.**
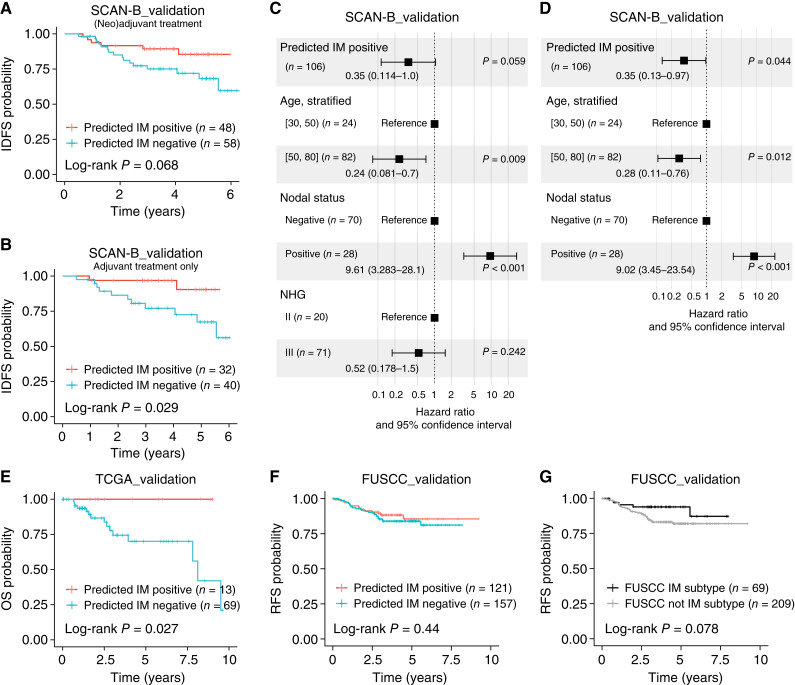
Association of the IM predictor with patient outcomes in independent TNBC cohorts. **A,** Kaplan–Meier plot of IDFS for predicted IM status for patients treated with neoadjuvant or adjuvant chemotherapy in the SCAN-B_validation cohort. **B,** Kaplan–Meier plot of IDFS for predicted IM status for patients treated with adjuvant chemotherapy only in the SCAN-B_validation cohort. **C,** Forest plot of multivariate Cox regression in the SCAN-B_validation cohort for patients treated with (neo)adjuvant chemotherapy using IDFS as endpoint. Covariates (including NHG) and HR with 95% confidence intervals shown. For groups, “]” means included in interval and “(“ means not included in interval. **D,** Forest plot as in (**C**), excluding NHG as a covariate in the multivariate Cox model. **E,** Kaplan–Meier plot of OS for predicted IM status in the TCGA_validation cohort. **F,** Kaplan–Meier plot of relapse-free survival for predicted IM status in the FUSCC_validation cohort for chemotherapy-treated patients. **G,** Kaplan–Meier plot of relapse-free survival in the FUSCC_validation cohort for the FUSCC IM subtype vs. all other FUSCC subtypes as reported in the original study for chemotherapy-treated patients. RFS, relapse-free survival.

To investigate the prognostic associations of the IM subtype predictor in independent cohorts, we performed survival analysis in the SCAN-B_validation, TCGA_validation, and FUSCC_validation cohorts. In both the SCAN-B_validation and FUSCC_validation cohorts, survival analyses were restricted to patients with records of (neo)adjuvant chemotherapy treatment. The SCAN-B_validationNAC cohort was not used for survival analysis because of short follow-up time for many patients. For the TCGA_validation cohort, treatment data were not readily available, and thus, survival analysis was performed for the total cohort using OS as clinical endpoint. Figure 4 shows the resulting Kaplan–Meier plots for the three cohorts using respective clinical endpoints, as well as multivariate Cox regression analyses for the SCAN-B_validation cohort.

In the SCAN-B_validation cohort, predicted IM status was borderline nonsignificant (log-rank test *P* = 0.068) in the complete chemotherapy-treated cohort, whereas it was statistically significant for patients with adjuvant treatment (i.e., excluding NAC-treated patients, log-rank test *P* = 0.029; Fig. 4A and B). Multivariate Cox regression showed a borderline nonsignificant association between predicted IM status and IDFS for a model including lymph node status, stratified patient age, and NHG status (*P* = 0.059; Fig. 4C). Excluding the nonsignificant NHG variable from the multivariate model made predicted IM status statistically significant (*P* = 0.044; Fig. 4D). In the full TCGA_validation cohort, predicted IM status was statistically significant using OS as clinical endpoint (log-rank test *P* = 0.027; Fig. 4E), whereas predicted IM status was not significant in the chemotherapy-treated subset of the FUSCC_validation cohort using recurrence-free survival as clinical endpoint (log-rank test *P* = 0.44; Fig. 4F). Notably, neither the IM subtype originally reported by Jiang and colleagues in FUSCC (7) nor the consensus IM class derived in this study was statistically significant in the FUSCC_validation chemotherapy-treated patient subset (Fig. 4G; log-rank test *P* = 0.078 and *P* = 0.21, respectively). To further elaborate on the difference in the FUSCC_validation cohort, we compared the classification overlap for tumors with IM classifications from all three predictors (consensus IM class, predicted IM class, and FUSCC IM subtype), finding that the FUSCC IM subtype matches an IM-positive classification by both the predicted (90.4% of FUSCC IM, two-sided *χ*^2^ test *P* = 8e−21) and consensus (89%, two-sided *χ*^2^ test *P* = 4e−24) IM methods to a large extent.

### Association of the IM predictor with treatment response in NAC patients with TNBC

**Figure 5.**
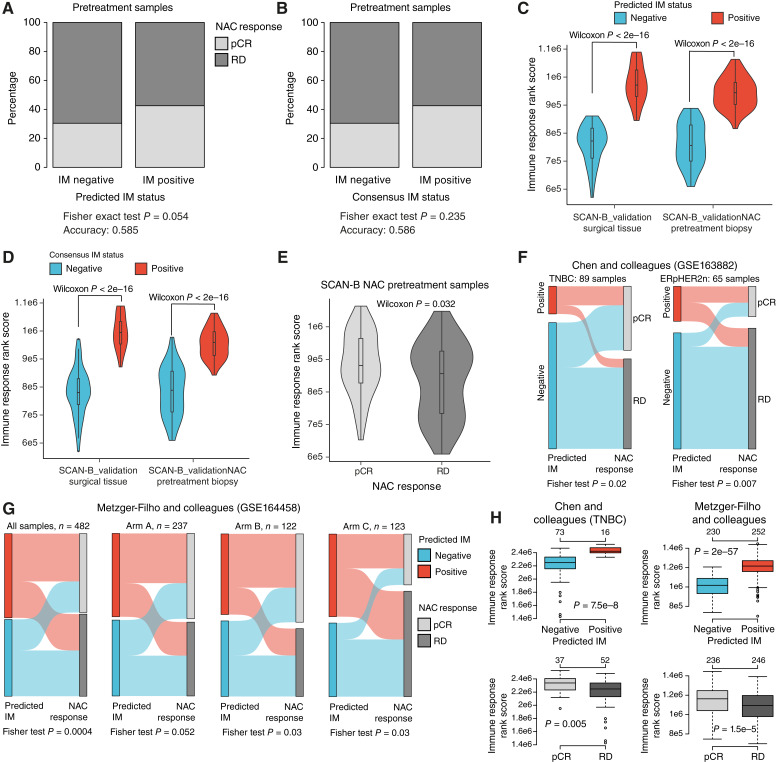
Association of the IM predictor with response to NAC. **A,** NAC treatment response (pCR/RD) vs. predicted IM subtype in pretreatment biopsies from 116 NAC-treated patients in the SCAN-B_validationNAC cohort. **B,** NAC treatment response vs. consensus IM subtype in pretreatment biopsies from the SCAN-B_validationNAC cohort. **C,** Violin plot of rank scores for an immune response metagene vs. predicted IM subtype in the 116 pretreatment biopsies. Corresponding rank scores for nonoverlapping tumors from the SCAN-B_validation cohort stratified by predicted IM subtype are shown as reference. **D,** Violin plot of rank scores for the immune response metagene vs. consensus IM labels in the 116 pretreatment biopsies. Corresponding rank scores for nonoverlapping tumors from the SCAN-B_validation cohort stratified by consensus IM subtype are shown as reference. **E,** Immune response metagene rank scores vs. NAC treatment response in the 116 pretreatment biopsies. **F,** Sankey plots of predicted IM status and NAC response in patients with TNBC (left) and patients with ER-positive and HER2-negative tumors (right) from Chen and colleagues (GSE163882; ref. 36). Association of predicted IM status and NAC response was tested using a Fisher exact test with *P* values shown for each group. **G,** Sankey plots of predicted IM status and NAC response in patients with TNBC from Metzger-Filho and colleagues (GSE164458; ref. 38) in total and stratified by treatment arm. Association of predicted IM status and NAC response was tested using a Fisher exact test, with *P* values shown for each group. **H,** Immune response metagene rank scores vs. predicted IM subtype in external NAC cohorts [Chen and colleagues (36) and Metzger-Filho and colleagues (38), top] and vs. corresponding NAC treatment response (bottom). For Chen and colleagues (36), only TNBC tumors are shown, whereas for Metzger-Filho and colleagues (38), all cases are included. Two-sided *P* values calculated using a Wilcoxon test. Numbers above boxplots correspond to sample sizes.

In addition to the prognostic evaluation of the IM classifier, we also investigated the treatment-predictive association of the IM classifier with response to NAC in the SCAN-B_validationNAC cohort, using RNA-seq profiles from 116 pretreatment diagnostic patient biopsies (*n* = 41 patients with pCR and *n* = 75 patients with RD). Predicted IM status was borderline nonsignificantly associated with treatment response, whereas the IM consensus class was even less associated with NAC response (Fisher exact test *P* = 0.054 and *P* = 0.235, respectively; Fig. 5A and B). To confirm that the IM classifier identified pretreatment core-needle tumor biopsies with elevated immune response as IM positive, we plotted rank-sum scores for the Fredlund and colleagues (44) immune response metagene versus predicted IM status and the IM consensus classes. Similar to the FUSCC_validation, TCGA_validation, and SCAN-B_validation cohorts, IM-positive (predicted as well as consensus-classified cases) core-needle tumor biopsies showed significantly higher immune response rank scores, equivalent with expression rank scores for surgically resected IM-positive tumors in the SCAN-B_validation cohort (Fig. 5C and D). This observation suggests that small pretreatment core-needle biopsies can be representative of the tumor TIME. Individual RNA-seq FPKM expression patterns for prototypical immune genes (*CD8A*, *PD-L1*, and *CD3D*) supported this observation (Supplementary Fig. S5C). Although tumors from NAC patients with pCR showed significantly higher immune response rank scores compared with patients with RD in pretreatment biopsies (two-sided Wilcoxon test *P* = 0.032), there was substantial overlap between the two score distributions (Fig. 5E).

To further test the association of the predicted IM status with treatment response, we analyzed two external NAC cohorts made available from Chen and colleagues (GSE163882; comprising different clinical subgroups, with *n* = 217 samples in total; ref. 36) and Metzger-Filho and colleagues (GSE164458; *n* = 482 TNBCs, the BrighTNess clinical phase III trial; refs. 37, 38). In Chen and colleagues (36), we found significant associations of predicted IM status with reported NAC response in patients with TNBC and in patients with ER-positive and HER2-negative tumors (two-sided Fisher exact test *P* = 0.02 and *P* = 0.007, respectively; Fig. 5F) but not in patients with HER2-positive tumors (two-sided Fisher exact test *P* = 1). In the large BrighTNess study, patients with stage II/III TNBC were randomized to receive NAC with paclitaxel, followed by doxorubicin/cyclophosphamide (treatment arm C, pCR rate 32.5%), or the same plus carboplatin (treatment arm B, pCR rate 56.6%) or carboplatin plus the PARP inhibitor veliparib concurrent with paclitaxel (treatment arm A, pCR rate 53.6%). In the total cohort, treatment arm B, and treatment arm C, we found a significant association of predicted IM status with NAC response (two-sided Fisher exact test, *P* < 0.05; Fig. 5G). In treatment arm A, the association was borderline nonsignificant (Fisher exact test *P* = 0.052; Fig. 5G). Similar to the SCAN-B_validationNAC cohort, we found that predicted IM-positive status in the two external TNBC NAC cohorts was significantly associated with elevated immune response rank scores (two-sided Wilcoxon test *P* < 1e−7), whereas there was a less strong association of these immune rank scores with NAC response illustrated by more overlapping rank score distributions (Fig. 5H).

### Changes in TIME status in paired pre- and posttreatment NAC patients

**Figure 6.**
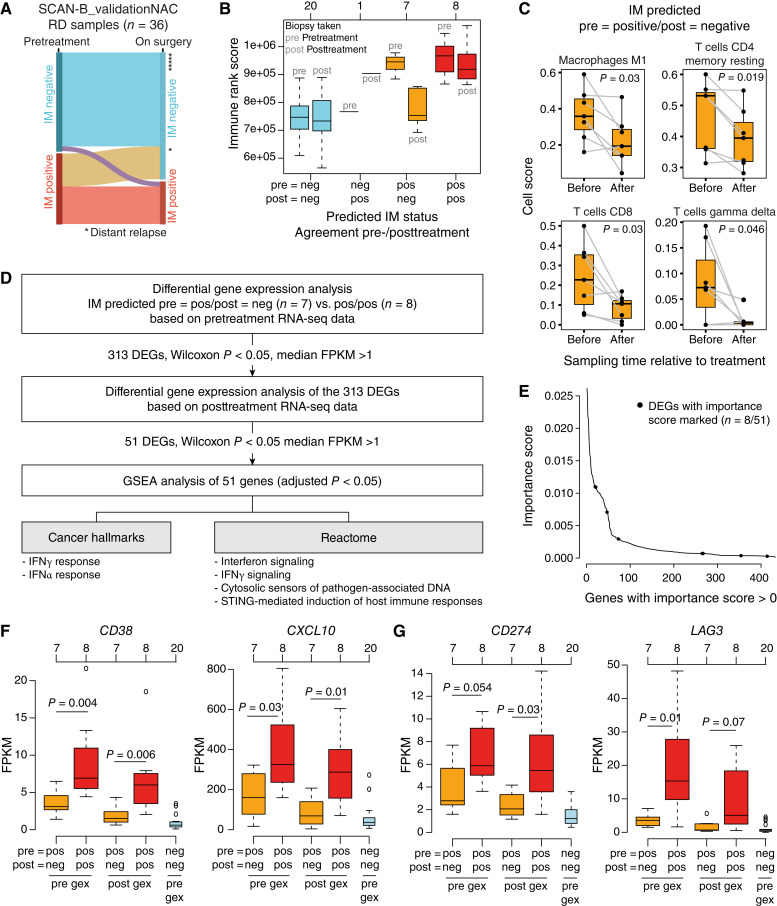
Changes in TIME status in paired pre- and posttreatment NAC patients. **A,** Sankey plot of predicted IM status before treatment and after treatment at surgery (based on RNA-seq data from surgical tissue) for 36 patients with RD and matched tumor specimens from the SCAN-B_validationNAC cohort, followed by a later recorded distant metastasis event (asterisks). **B,** Rank scores for the immune response metagene for the 36 RD patients with paired pre- and posttreatment samples stratified by their combined pre- and posttreatment IM prediction. Left: immune rank scores based on RNA-seq data from pretreatment biopsies. Right: immune rank scores based on RNA-seq data from posttreatment surgical tissue. Numbers above boxplots correspond to sample sizes. **C,** Immune cell type scores imputed with CIBERSORTx for cell types that differed between pre- and posttreatment samples for IM-predicted positive tumors before treatment that were considered IM negative after treatment. Unadjusted *P* values reported from two-sided paired *t* tests. **D,** Strategy to identify differentially expressed genes between predicted IM-positive tumors before treatment that changed to IM negative after treatment (pos/neg) vs. those that remained IM positive (pos/pos). **E,** Importance scores for the 433 genes in the IM predictor with scores > 0. Scores for the eight genes from the set of 51 identified in (**D**) overlapping with the 433 are marked by points. **F,** FPKM gene expression (gex) data for the interferon signaling–associated genes *CD38* (hallmark IFNγ response) and *CXCL10* (hallmark IFNα response) from pretreatment and posttreatment samples stratified by their predicted IM status. Two-sided *P* values calculated using a Wilcoxon test. Gene expression of each gene in IM-predicted negative tumors both before and after treatment (neg/neg) included for reference. **G,** Same as (**F**) but for the immune-inhibitory genes *PD-L1* (*CD274*) and *LAG3*. DEG, differentially expressed gene; GSEA, gene set enrichment analysis.

The availability of 36 paired surgical tumor tissue resections with RNA-seq data from patients with RD in the SCAN-B_validationNAC cohort allowed us to explore how predicted IM subtypes change from before (core needle) to after treatment (surgical specimen). Of the 36 paired samples, 21 tumors were predicted IM negative and 15 were predicted IM positive based on pretreatment core-needle biopsies. As shown in Fig. 6A, predicted IM-negative tumors typically remained IM negative after treatment (20 of 21, 95.2%), whereas 46.7% (seven of 15) of initially IM-positive tumors were predicted IM negative in posttreatment surgical tissue. Predicted IM status was consistent in the matched samples in that IM-positive tumors showed elevated rank scores of the immune metagene (Fig. 6B), providing preliminary support to the finding that the tumors changing IM status from before treatment to after treatment may have altered their TIME during treatment.

To further explore this hypothesis, we analyzed absolute cell fractions of 22 different immune cell types imputed through deconvolution of pretreatment gene expression (RNA-seq) data with CIBERSORTx for the predicted IM status agreement groups in Fig. 6B. Of the 22 cell types, we found a difference for M1 macrophages (two-sided paired *t* test *P* = 0.03), CD8-positive T cells (*P* = 0.03), resting memory CD4-positive T cells (*P* = 0.019), and gamma delta T cells (*P* = 0.046) in tumors that changed IM status from pretreatment positive to posttreatment negative (positive/negative), all moving from higher to lower cell fractions (Fig. 6C; Supplementary Fig. S6 for all cell types). To analyze this exploratory observation further, we performed differential gene expression between tumors with a positive/negative IM status versus tumors with a positive/positive IM status as outlined in Fig. 6D. The resulting final set of 51 genes was statistically enriched especially for interferon signaling pathways (Fig. 6D). Only eight of 51 genes were IM predictor genes with importance scores > 0, showing typically intermediate-to-lower scores (Fig. 6E). The expression of two of the interferon-associated genes not included in the IM predictor, *CD38* and *CXCL10*, is exemplified in Fig. 6F, showing a pattern of lower expression in positive/negative samples more consistent with IM-negative tumors. Interestingly, two key immune-inhibitory genes, *PD-L1* (*CD274*) and *LAG3*, showed similar patterns to *CD38* and *CXCL10* (Fig. 6G). Despite the borderline nonsignificant test results (two-sided Wilcoxon test *P* values between 0.054 and 0.08), these observations suggest further exploration in larger tumor cohorts as the current analyses are statistically limited by the small group sizes of the IM groups.

Finally, distant metastasis development information was also available for patients with pre- and posttreatment samples. We compared predicted IM status for the 36 RD patients with paired tumor specimens to check whether a patient developed a distant metastasis within the limited follow-up period of this study (illustrated in Fig. 6A). Here, 23.8% (five of 21) of patients with a predicted IM-negative tumor before treatment developed a metastasis compared with only 6.7% (one of 15) of patients with a predicted IM-positive tumor before treatment (two-sided *χ*^2^ test *P* = 0.36). A similar comparison based on IM prediction of the posttreatment surgical resection tissue showed that 22.2% (six of 27) of patients with a predicted IM-negative tumor after treatment developed a distant metastasis compared with 0% (zero of nine) for patients with a predicted IM-positive tumor after treatment (two-sided *χ*^2^ test *P* = 0.30).

## Discussion

The clinical relevance of the TIME for prognostication and treatment prediction in primary TNBC has repeatedly been reported. In this study, we derived a gene expression–based stand-alone predictor of immune infiltration applicable to individual samples based on the original IM subtype proposed by Lehmann and colleagues (5). The rationale for our approach was (i) the repeated observation of this mRNA phenotype in different TNBC studies, (ii) the strong association of the optimized consensus IM subtype classification with patient outcomes in patients treated with adjuvant chemotherapy in the population-based training cohort, and (iii) the practical limitation of the current IM classifier tool by the inherent bias of NC classifiers to, e.g., cohort composition that could be solved by a stand-alone predictor. Notably, the non-random perturbation of the cohort composition shown in Fig. 1 will affect not only IM status but also TNBCtype subtype assignments to the proposed basal-like 1, basal-like 2, mesenchymal, and luminal androgen receptor subtypes. It should be acknowledged that many other training labels (e.g., quantiles of immune gene rank scores, expression of individual immune-associated genes, or even TIL-based training labels) that correlate with the IM subtype class could have been used instead, likely generating highly similar end results.

The derived IM predictor showed prediction agreement between 78% and 91% in independent RNA-seq–profiled TNBC cohorts of different patient demographics and ethnicity. Notably, although not all tumors in a cohort can be subtyped by the TNBCtype tool (see e.g., the FUSCC_validation cohort in Table 1), all tumors can be subtyped by the stand-alone predictor with consistent rank scores of the immune response metagene (Supplementary Fig. S7). It should be noted that the IM prediction agreement in this context is based on consensus-derived labels from the TNBCtype webtool, which is inherently biased depending on the sample cohort to classify, generating similar IM class proportions in each cohort. Thus, achieving 100% agreement is likely difficult due to the inherent issues with NC classifiers applied to different cohorts. A more relevant validation of the IM predictor may thus be related to agreement with different immune response features and patient outcomes on a group level. Based on comparisons of consensus and predicted IM subtypes versus TIL estimates, *in situ* IHC immune cell marker counts, and mRNA-derived immune metagene rank scores, we demonstrate that the original IM subtype classification, as well as the developed classifier, can identify TNBCs with higher and lower immune infiltration. Moreover, tumors with discordant IM class show intermediate immune rank scores compared with tumors with concordant classifications, indicating that these likely represent borderline cases in the unimodal distribution of tumor immune response levels. As such, we propose that the predicted IM and original IM subtypes should be regarded as surrogates for generally higher or lower immune infiltration. Supporting this claim, we found that *in situ* cell counts of different immune cell markers were all higher in IM-positive treatment-naïve SCAN-B_training tumors. In addition, genes regarded as having importance scores > 0 in the final predictor model were heavily enriched for different immune response–associated processes, suggesting that the derived model is likely mainly driven by tumor-extrinsic expression patterns (i.e., expression in nonmalignant cells) as also seen through analyses of TNBC cell lines and scRNA-seq data from both TNBC tumors and normal breast tissue. To further analyze the immune response nature of the IM classifier, we performed a TCR/BCR repertoire analysis using MiXCR based on bulk tumor RNA-seq data, finding that TCR genes (*TRA* and *TRB*), but not BCR genes, showed higher diversity in IM-positive tumors (and thus also in tumors with high TIL counts). This finding aligns with a general concept of higher receptor diversity in previously untreated immune-“warm” TNBC tumors. To assess the prognostic performance of the derived predictor, we performed survival analyses in three independent RNA-seq cohorts totaling 519 patients. Strongest prognostic associations were observed in the TCGA_validation and SCAN-B_validation cohorts, especially in the subgroup of adjuvant-treated patients in the SCAN-B cohort, which is equivalent to the patient group in which the predictor was developed (i.e., showing similar demographic context, clinical management, and treatment context). In contrast, although we observed association of the IM subtype with rank scores of the immune response metagene (for consensus as well as predicted IM subtypes) in the FUSCC_validation cohort of East Asian ethnicity, the prognostic association is less strong, consistent with the original study’s own immune subtype also not being significantly associated with patient outcomes. Notably, in the FUSCC_validation cohort, the reported FUSCC immune subtype comprised 25% of all tumors compared with our computed consensus IM proportion of 38% IM-positive cases in tumors possible to classify through the online TNBCtype tool. Together, the results in the FUSCC_validation cohort suggest an impact of either ethnicity, treatment regimens, or cohort selection on the conflicting results compared with the TCGA_validation and SCAN-B_validation cohorts.

To investigate the treatment-predictive association of the IM classifier for NAC, we first applied it to a cohort of 116 NAC-treated (mainly preoperative EC + taxane chemotherapy) patients with TNBC from SCAN-B (SCAN-B_validationNAC cohort) with consensus IM classes. With conventional NAC, 30% to 40% of patients typically achieve pCR (53), consistent with an observed pCR rate of 35.3% in the SCAN-B_validationNAC cohort. In this cohort, we found that neither the IM predicted (borderline nonsignificant) nor the IM consensus class was significantly associated with treatment response (pCR/RD) despite a similar association with a more immune-“warm” phenotype as in surgical tumor tissue. Reasons for this could be several, including e.g., tuning of the predictor and composition of the patient cohort assigned to NAC. Notably, the SCAN-B_validationNAC cohort comprises patients from a population-representative enrollment based on national treatment guidelines at the time of diagnosis. These patients would thus typically have features of more aggressive disease and likely an intrinsically worse prognosis. In contrast to the SCAN-B_validationNAC cohort, significant associations of the predictor with treatment response were observed in two external NAC cohorts, including the large BrighTNess clinical phase III study with three different treatment arms (37). Only in one treatment arm was the association borderline nonsignificant, similar to the SCAN-B_validationNAC cohort. However, as shown in Fig. 5, although patients with pCR in all NAC cohorts had significantly higher rank scores of an immune metagene in pretreatment biopsies compared with pretreatment biopsies from patients with RD, substantial overlap between the distributions exists. Together, this suggests a challenging classification task of using only immune response–associated genes. Indeed, different transcriptional programs involving immune, cell cycle/mitotic, and RNA splicing–related pathways have been suggested to be significantly associated with favorable clinical outcomes in TNBC (54).

To further explore how the IM predictor could be used to classify the TIME in NAC-treated patients, we focused on patients with RD and matched pretreatment core-needle biopsy tissue and posttreatment surgical tumor tissue. Importantly, patients with RD after NAC represent an important clinical group because of a high risk of distant relapse (55–57). In these patients, we observed that the predicted IM status changed in 22.2% of cases, involving in all but one case of a shift from a pretreatment predicted IM-positive status to an IM-negative status after treatment, suggesting a potential reshaping of the TIME during NAC to a more immune-“cold” environment in some tumors. Consistently, previous studies have reported indications of immune depletion in posttreatment specimens as one component of the tumor reshaping during NAC treatment pressure (57, 58). To explore this shift in IM status, we used RNA-seq immune cell deconvolution, finding that in initially immune-“warm” tumors turning “cold,” absolute cell scores of pro-inflammatory M1 macrophages decreased, which also happened for three types of T cells. Exploratory differential gene expression identified lower expression of interferon signaling pathway genes in the tumors turning cold already before treatment, as well as similar expression patterns of typical immune-inhibitory genes like *PD-L1* and *LAG3*. These differential expression patterns are in contrast to the similar general immune response level before treatment. Together, these preliminary results obtained from this small cohort suggest the existence of potentially different pretreatment TIMEs that may respond differently to NAC, warranting further confirmation in larger matched tumor cohorts.

The analysis of matched pre- and post-tumor samples for patients with RD also revealed that a predicted IM-positive status based on RNA-seq data in this challenging high-risk group of patients seems to be a potential marker of better long-term outcomes. Although one RD patient with an IM-positive predicted diagnostic core-needle biopsy developed a recurrence, zero patients with a predicted IM-positive tumor after treatment did so. If validated in larger series, an IM-positive posttreatment prediction could represent a biomarker for good long-term prognosis in this high-risk patient group. However, far from all patients with RD develop distant relapse, suggesting a more complex molecular risk pattern in NAC patients besides an immune-“cold” TIME. Indeed, alternative prognostic and predictive factors have been proposed in TNBC, e.g., homologous recombination deficiency (26, 59, 60) and other genomic alterations in NAC-treated patients specifically (54, 57, 61). To address the high-risk RD patient group, conceivable approaches could involve creating combined predictors (including multiple transcriptional programs as well as other data types) trained on treatment response or distant relapse in sufficiently large tumor cohorts.

Limitations of the study include that the current IM classifier has been developed for RNA-seq FPKM values comprising a large number of genes. However, only a limited set of genes had positive importance scores representing the key gene set to be measured. Potential adaptation to other expression measurements (e.g., transcripts per million or counts) and technologies limited in panel size but applicable to degraded RNA could mean retraining the predictor, e.g., with a restricted gene set. Interestingly, we observed that the FPKM-based predictor provided clinically relevant results when applied to reads per kilobase million data from GSE164458, suggesting some preliminary generalizability. We also note that the highest classification agreement was observed in the SCAN-B_validation cohort, a cohort that is sequenced using similar protocols and facilities but that is also of the same population-representative demographic context as the original training cohort. It should thus be acknowledged that training and calibration (cutoffs for IM status) in other cohorts may affect predictor performance, stressing the importance of representative patient cohorts for predictor training. Retraining the predictor using an alternative cutoff for IM status or other immune-related training labels is, however, straightforward. Moreover, several of the included prognostic RNA-seq validation cohorts are still relatively small and, apart from the SCAN-B_validation cohort, without detailed chemotherapy treatment data, stressing the need for additional validation in larger cohorts. Although the treatment landscape of primary TNBC is evolving with the introduction of combined NAC and ICT (62), it should be noted that there is still no consensus about treatment-predictive biomarkers (like TILs or PD-L1 expression) for ICT (63) as treatment effect has been observed independent of, e.g., PD-L1 status (22, 64). As such, PD-L1 expression is currently not included as a treatment-predictive marker in guidelines for preoperative ICT. Importantly, publicly available mRNA-profiled TNBC cohorts treated with either neoadjuvant or adjuvant ICT are currently very scarce. As a result, the treatment-predictive and prognostic potential of the developed IM predictor with respect to ICT remains to be explored. Finally, to improve predictor performance, it may be beneficial to develop integrated models that include not only RNA-seq data but also clinicopathologic information available at the time of diagnosis.

In conclusion, we provide an RNA-seq–based stand-alone predictor of immune response association in TNBC based on the proposed IM subtype by Lehmann and colleagues (5). This predictor allows individual tumors to be classified based only on their FPKM estimates, representing a simplification compared with existing options. Moreover, it enables the classification of smaller cohorts with compositions that differ from those in the original Lehmann study. We demonstrate that predicted IM-positive tumors are associated with elevated infiltration of TILs and other immune cells, higher expression of genes associated with immune response, higher diversity of TCR genes, better NAC treatment response, and finally better prognosis in independent cohorts. Given recent studies showing that patients with TNBC with chemo-naïve, immune-warm, node-negative tumors have an excellent long-term prognosis, the concept of chemotherapy de-escalation has been proposed and could now be considered for prospective clinical trials (10). Here, the availability of stand-alone classifiers for mRNA-based prediction may be of importance for enhancing the utility of, e.g., RNA-seq–based analyses for translational endpoints in (neo)adjuvant clinical trials, particularly those aimed at optimizing the use of chemotherapy and, more importantly, ICT. Ultimately, RNA-seq–based analysis may serve as a multipurpose assay for diagnostics and treatment decision support in early breast cancer by providing benchmarked equivalent estimates of current clinical markers, molecular subtypes, and risk assessments as recently demonstrated in the SCAN-B study (30).

## Supplementary Material

Supplementary Table 1 Supplementary Table 1 providing annotations for included samples.

Supplementary Table 2 Supplementary Table 2 (RNAseq estimates)

Supplementary Table 3 Supplementary Table 3. Predictor genes and importance scores.

Supplementary Figure 1 Supplementary Figure 1 showing definition of IM subtype correlation cut-off based on patient outcome

Supplementary Figure 2 Supplementary Figure 2 showing IM predictor development and performance in the training cohort.

Supplementary Figure 3 Supplementary Figure 3 showing expression in different non-malignant cells of genes with highest importance score for the final random forest model.

Supplementary Figure 4 Supplementary Figure 4 showing IM predictor probabilities in independent validation cohorts.

Supplementary Figure 5 Supplementary Figure 5 showing TCR/BCR diversity and immune response association of the IM predictor in independent TNBC cohorts.

Supplementary Figure 6 Supplementary Figure 6 showing immune cell fraction estimates for samples obtained pre- and post-treatment.

Supplementary Figure 7 Supplementary Figure 7 showing immune metagene rank scores for the IM classifier applied to 23 FUSCC_validation tumors with no IM consensus label from the online TNBCtype tool.

## Data Availability

The genomic datasets supporting the conclusions of this article are available in open repositories as described in the original studies or as supplementary data. Briefly, all SCAN-B RNA-seq data used in this study are available from https://data.mendeley.com/datasets/yzxtxn4nmd/3 as originally reported by Staaf and colleagues (30), except for the FPKM data for the paired NAC SCAN-B cohort which are available as Supplementary Table S2. The RNA-seq data for the FUSCC cohort used in this study were originally reported by Jiang and colleagues (7) and are available through the Sequence Read Archive (https://www.ncbi.nlm.nih.gov/bioproject/PRJNA486023). The TCGA data used in this study are available from the Genomic Data Commons data portal (https://portal.gdc.cancer.gov). Gene expression data from TNBC cell lines reported by Jovanović and colleagues (39) used in this study are available from GEO under accession number GSE202770 (https://www.ncbi.nlm.nih.gov/geo/query/acc.cgi?acc=GSE202770). Gene expression data from Chen and colleagues (36) used in this study are available from GEO under accession number GSE163882 (https://www.ncbi.nlm.nih.gov/geo/query/acc.cgi?acc=GSE163882). Gene expression data from Loibl and colleagues (37, 38) used in this study are available from GEO under accession number GSE164458 (https://www.ncbi.nlm.nih.gov/geo/query/acc.cgi?acc=GSE164458). The scRNA-seq TNBC data used in this study were compiled by Pang and colleagues (41) and are available from https://doi.org/10.6084/m9.figshare.25019903. The reported scRNA-seq data of normal breast tissue from Reed and colleagues (42) used in this study are deposited in the CELLxGENE platform and available from https://cellxgene.cziscience.com/collections/48259aa8-f168-4bf5-b797-af8e88da6637. All analyses were performed using open-source software such as the R statistical language and Python. The stand-alone IM classifier is publicly available at https://github.com/StaafLab/PredImm.
