# Supplementary material for: Stand-alone Transcriptional Immune Response Prediction in Primary Triple-Negative Breast Cancer
Source: Cancer Res Commun. 2025 Dec 15;5(12):2157–74. doi: 10.1158/2767-9764.CRC-25-0453 (PMC12703016; doi:10.1158/2767-9764.CRC-25-0453)
Supplement: Supplementary Figure 1 — showing definition of IM subtype correlation cut-off based on patient outcome [file crc-25-0453_supplementary_figure_1_suppsf1.pdf]

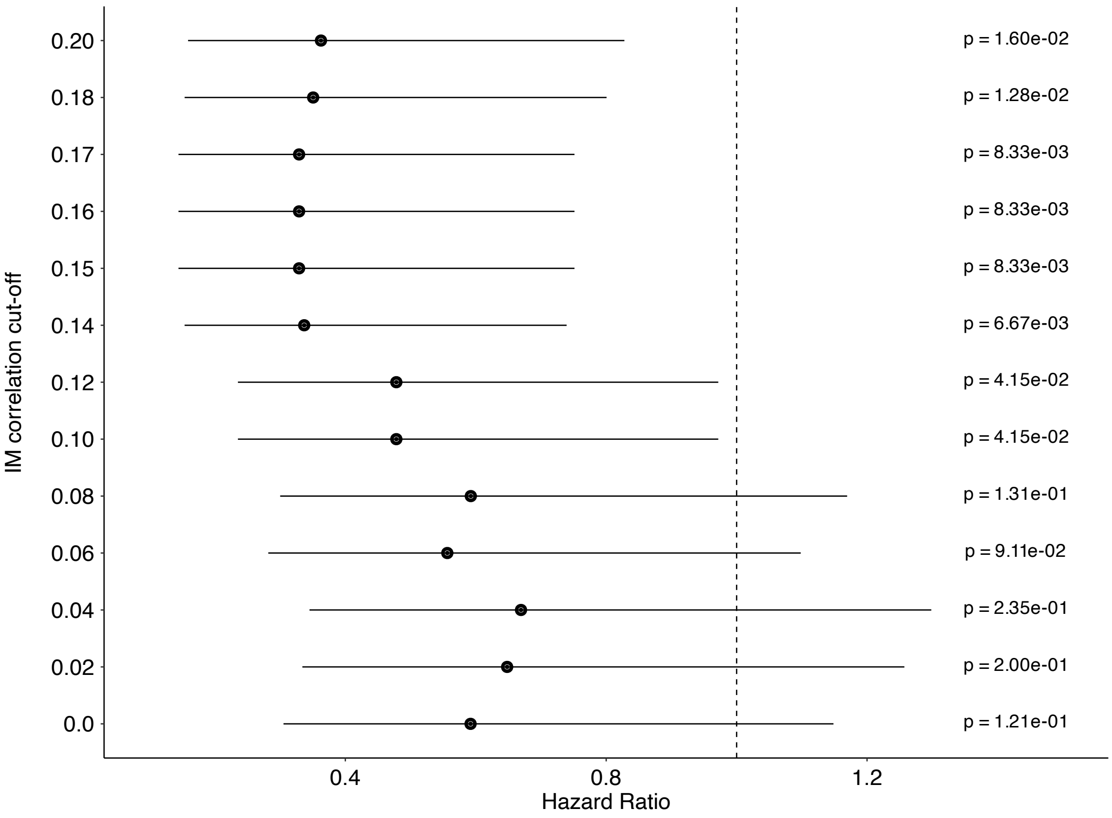

Supplementary Figure 1. Definition of IM subtype correlation cut-off based on patient outcome. Univariate Cox regression results (hazard ratios, 95% confidence intervals and p-values), using invasive disease-free survival as clinical endpoint in patients with adjuvant chemotherapy, for different correlation cut-offs to form IM-positive and IM-negative consensus groups in the SCAN-B_training cohort. Correlation per tumor to the IM-subtype centroid was obtained from the online TNBCtype tool.
