# Supplementary material for: Stand-alone Transcriptional Immune Response Prediction in Primary Triple-Negative Breast Cancer
Source: Cancer Res Commun. 2025 Dec 15;5(12):2157–74. doi: 10.1158/2767-9764.CRC-25-0453 (PMC12703016; doi:10.1158/2767-9764.CRC-25-0453)
Supplement: Supplementary Figure 2 — showing IM predictor development and performance in the training cohort. [file crc-25-0453_supplementary_figure_2_suppsf2.pdf]

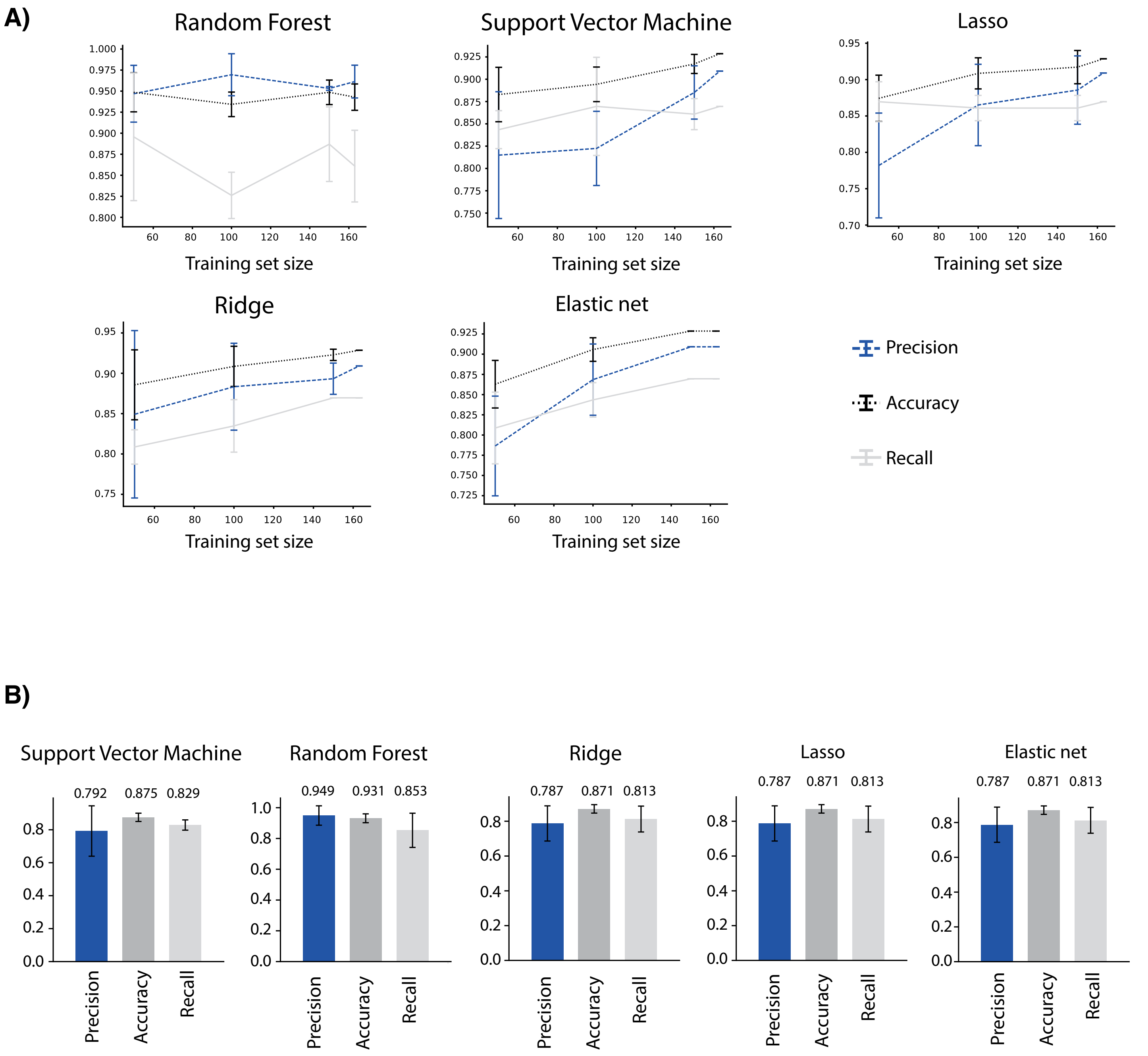

Supplementary Figure 2. Predictor development and performance in the training cohort. A) Mean precision, accuracy, and recall rate for Random Forest, Support Vector Machine, Logistic Regression with lasso regularisation, Logistic Regression with ridge regularisation, and Logistic Regression with Elastic net regularisation in the SCAN-B_training cohort over increasing training set sizes using a fixed validation set. **B)** Mean precision, accuracy, and recall rate based on 5-fold cross validation for Support Vector Machine, Random Forest, Logistic Regression with lasso regularisation, Logistic Regression with ridge regularisation, and Logistic Regression with Elastic net regularisation in the SCAN-B_training cohort.
