# Supplementary material for: Stand-alone Transcriptional Immune Response Prediction in Primary Triple-Negative Breast Cancer
Source: Cancer Res Commun. 2025 Dec 15;5(12):2157–74. doi: 10.1158/2767-9764.CRC-25-0453 (PMC12703016; doi:10.1158/2767-9764.CRC-25-0453)
Supplement: Supplementary Figure 3 — showing expression in different non-malignant cells of genes with highest importance score for the final random forest model. [file crc-25-0453_supplementary_figure_3_suppsf3.pdf]

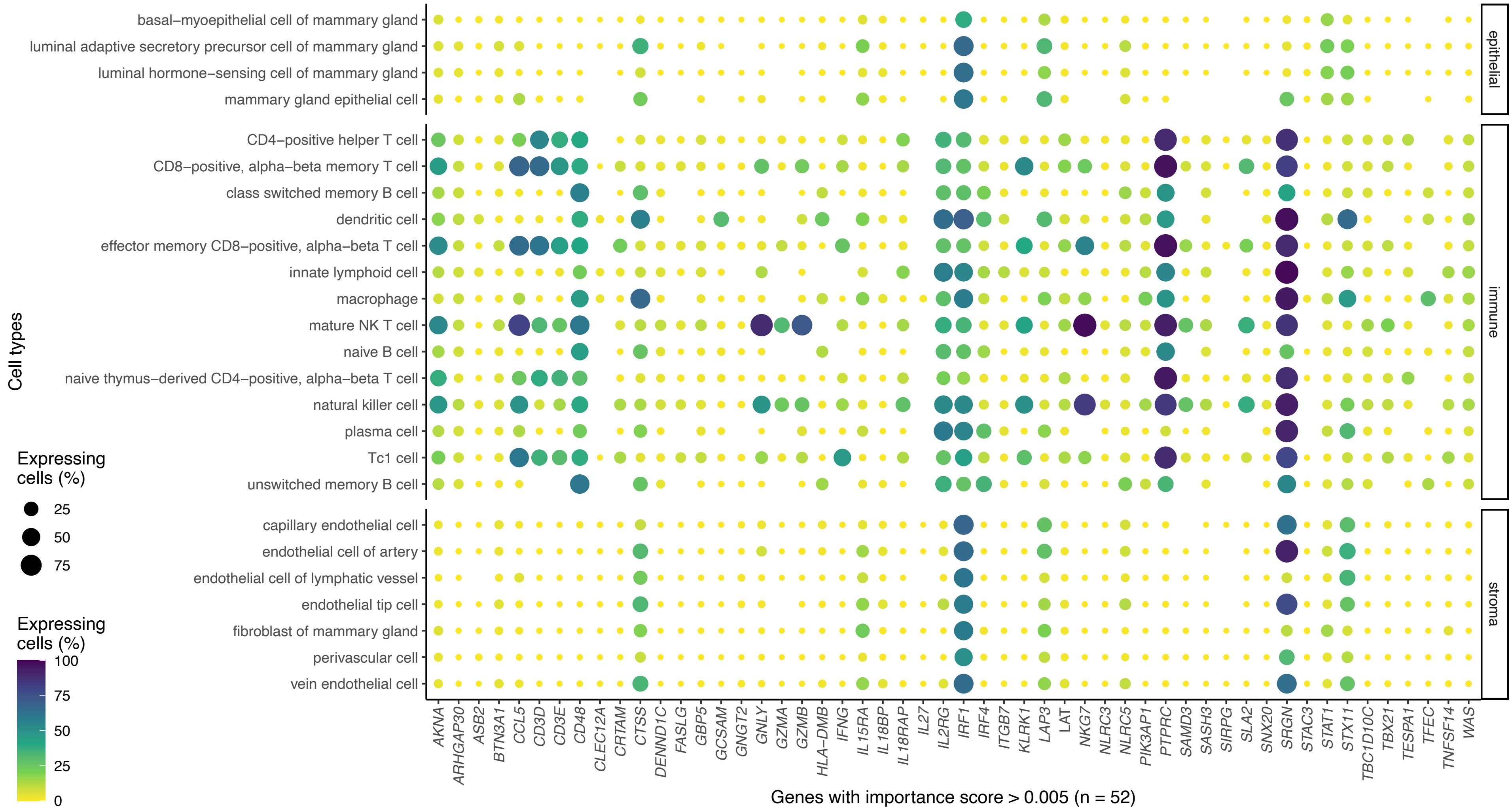

Supplementary Figure 3. Expression in different non-malignant cells of genes with highest importance score for the final random forest classifier. Percentages of several cell types expressing a given gene calculated from an initial pool of 803,283 cells from the epithelial, immune, and stromal compartments made available from Reed AD, et al., 2024. A cell was counted as expressing a gene whenever its RNAseq count was above zero.
