# Supplementary material for: Stand-alone Transcriptional Immune Response Prediction in Primary Triple-Negative Breast Cancer
Source: Cancer Res Commun. 2025 Dec 15;5(12):2157–74. doi: 10.1158/2767-9764.CRC-25-0453 (PMC12703016; doi:10.1158/2767-9764.CRC-25-0453)
Supplement: Supplementary Figure 4 — showing IM predictor probabilities in independent validation cohorts. [file crc-25-0453_supplementary_figure_4_suppsf4.pdf]

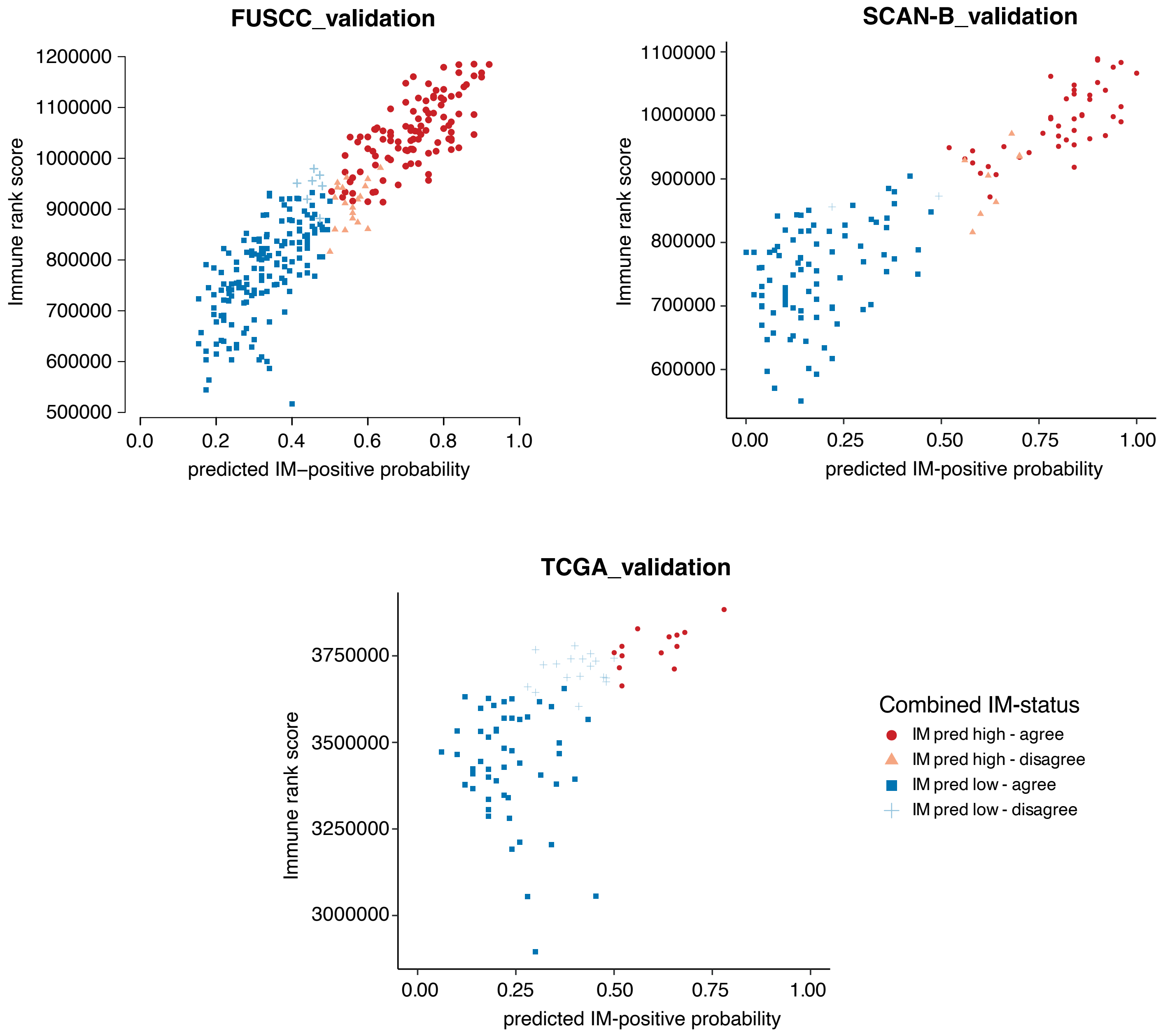

Supplementary Figure 4. Predictor probabilities in independent validation cohorts. Scatter plots of the predicted probability for IM-positive versus rank scores for an immune response associated metagene in the FUSCC_validation, SCAN-B_validation, and TCGA_validation cohorts. Tumours are coloured for a 4-tier label derived from combination of IM consensus and predicted IM labels.
