# Supplementary material for: Stand-alone Transcriptional Immune Response Prediction in Primary Triple-Negative Breast Cancer
Source: Cancer Res Commun. 2025 Dec 15;5(12):2157–74. doi: 10.1158/2767-9764.CRC-25-0453 (PMC12703016; doi:10.1158/2767-9764.CRC-25-0453)
Supplement: Supplementary Figure 5 — showing TCR/BCR diversity and immune response association of the IM predictor in independent TNBC cohorts. [file crc-25-0453_supplementary_figure_5_suppsf5.pdf]

A)

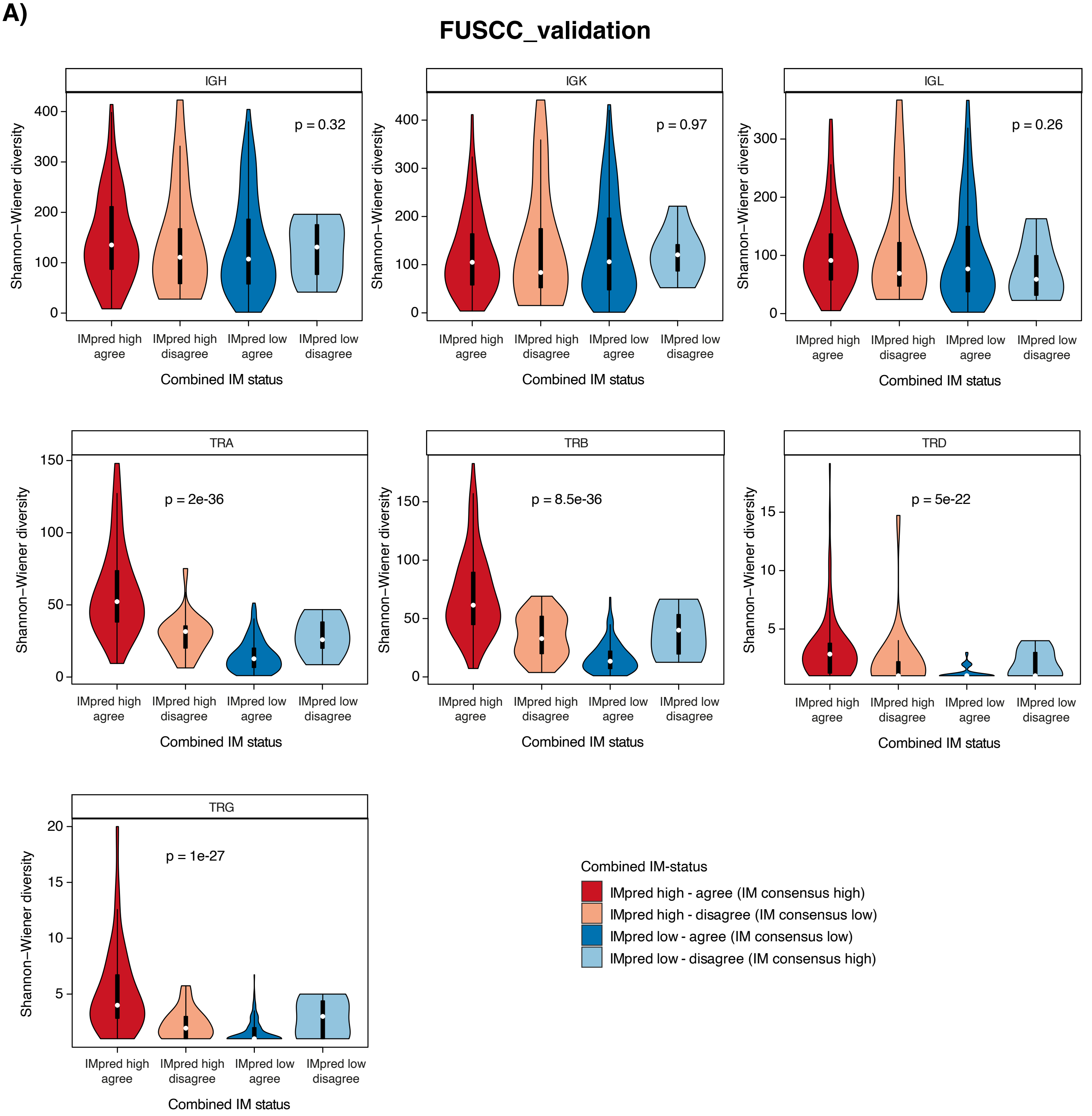

Supplementary Figure 5. TCR/BCR diversity and immune response association of the IM predictor in independent TNBC cohorts. Violin plots of Shannon-Wiener diversity score based on Mixcr analyses of tumour RNAseq data for T-cell (*TRA*, *TRB*, *TRD*, *TRG*) and B-cell (*IGH*, *IGK*, *IGL*) receptor genes versus a 4-tier label derived from combination of IM consensus and predicted IM labels in the **A)** FUSCC_validation and **B)** SCAN-B_validation cohorts. **C)** FPKM expression of different immune response associated genes, *MKI67* (Ki67) and steroid response genes for SCAN-B_validationNAC pre-treatment biopsy specimens and SCAN-B_validation surgical tissue specimens stratified by predicted IM-status. In A and B, two-sided p-values were calculated using Kruskal-Wallis test.

B)

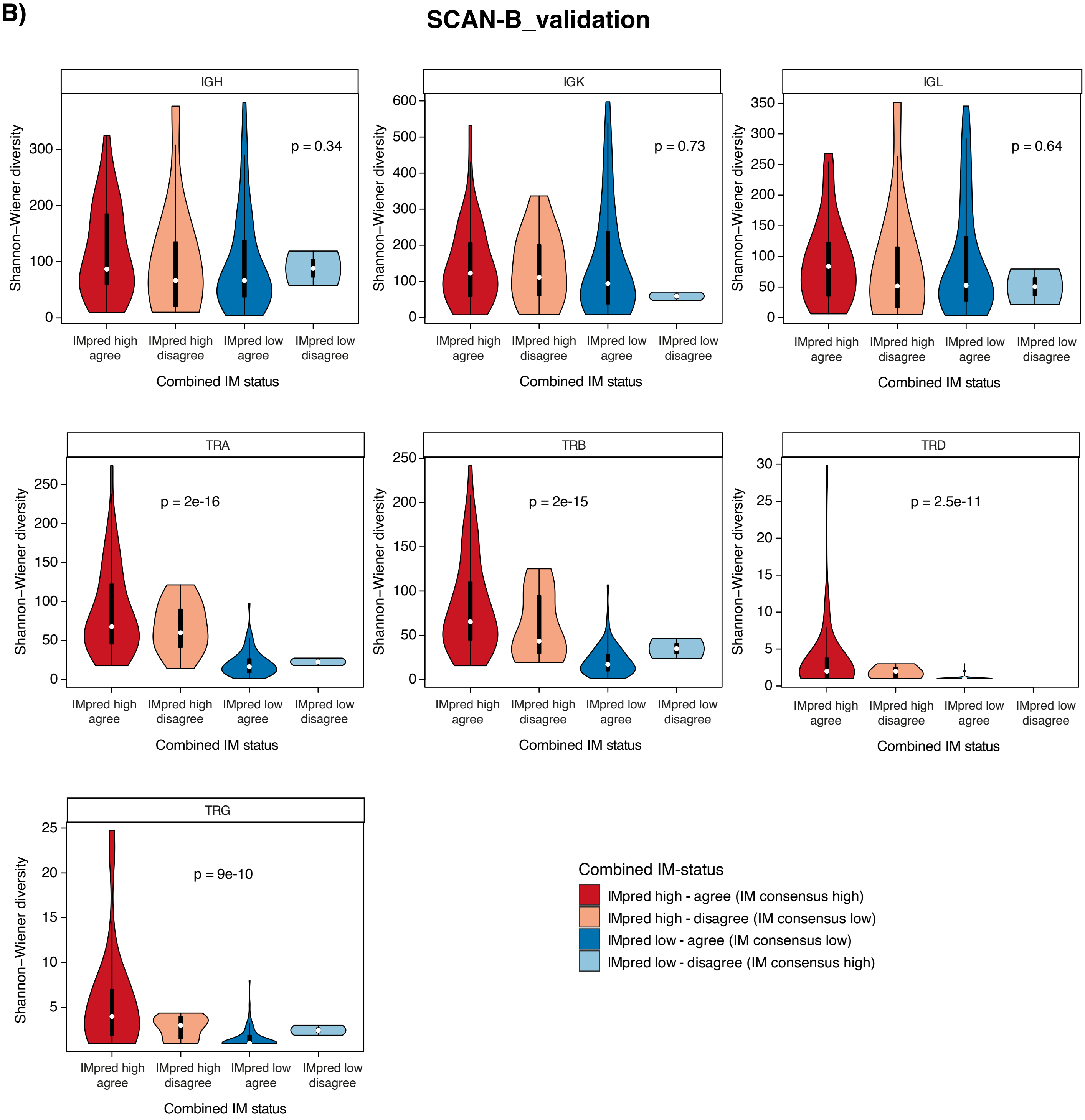
SCAN-B_validation
IGH
IGK
IGL
TRA
TRB
TRD
TRG
Shannon−Wiener diversity
Combined IM status
IMpred high agree
IMpred high disagree
IMpred low agree
IMpred low disagree
p = 0.34
p = 0.73
p = 0.64
p = 2e-16
p = 2e-15
p = 2.5e-11
p = 9e-10
Combined IM-status
IMpred high - agree (IM consensus high)
IMpred high - disagree (IM consensus low)
IMpred low - agree (IM consensus low)
IMpred low - disagree (IM consensus high)

C)

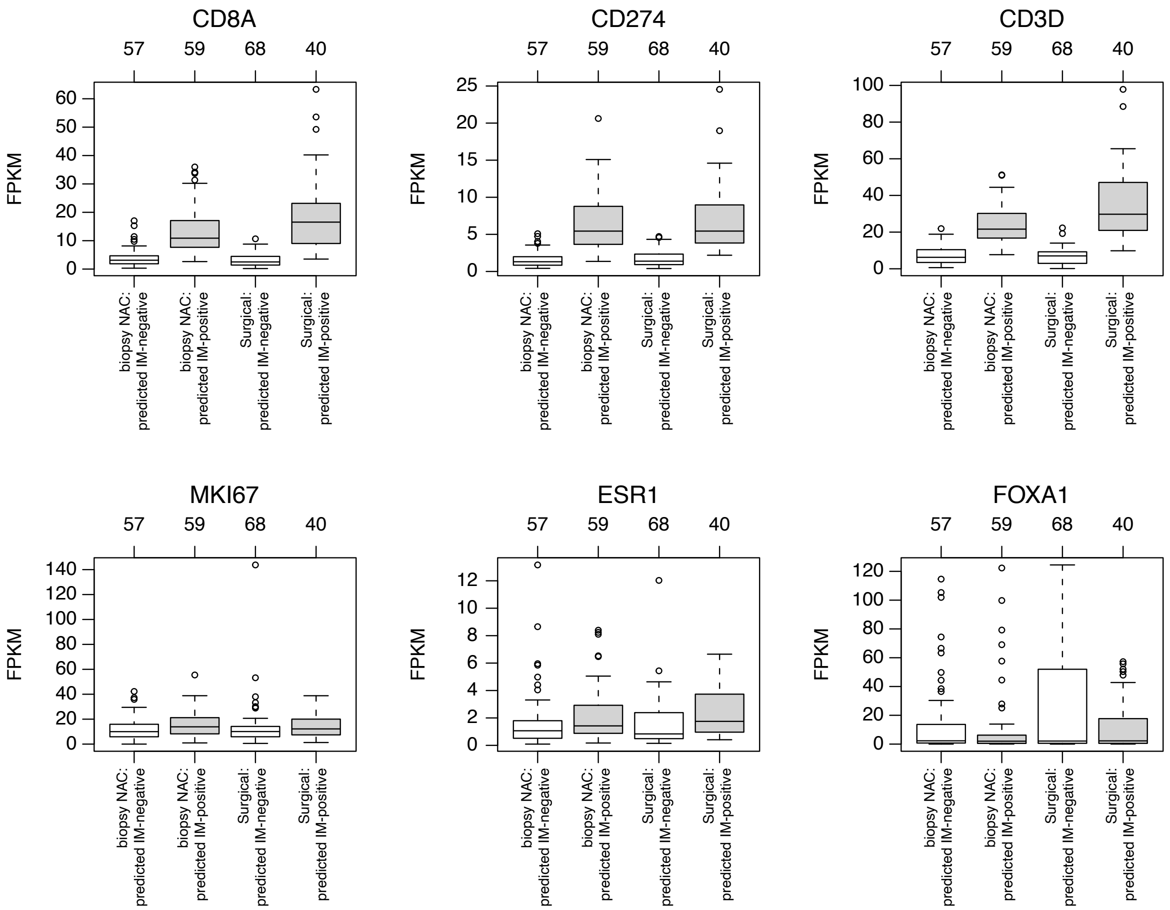

CD8A
CD274
CD3D
MKI67
ESR1
FOXA1
57
59
68
40
FPKM
biopsy NAC: predicted IM-negative
biopsy NAC: predicted IM-positive
Surgical: predicted IM-negative
Surgical: predicted IM-positive
