# Supplementary material for: Stand-alone Transcriptional Immune Response Prediction in Primary Triple-Negative Breast Cancer
Source: Cancer Res Commun. 2025 Dec 15;5(12):2157–74. doi: 10.1158/2767-9764.CRC-25-0453 (PMC12703016; doi:10.1158/2767-9764.CRC-25-0453)
Supplement: Supplementary Figure 6 — showing immune cell fraction estimates for samples obtained pre- and post-treatment. [file crc-25-0453_supplementary_figure_6_suppsf6.pdf]

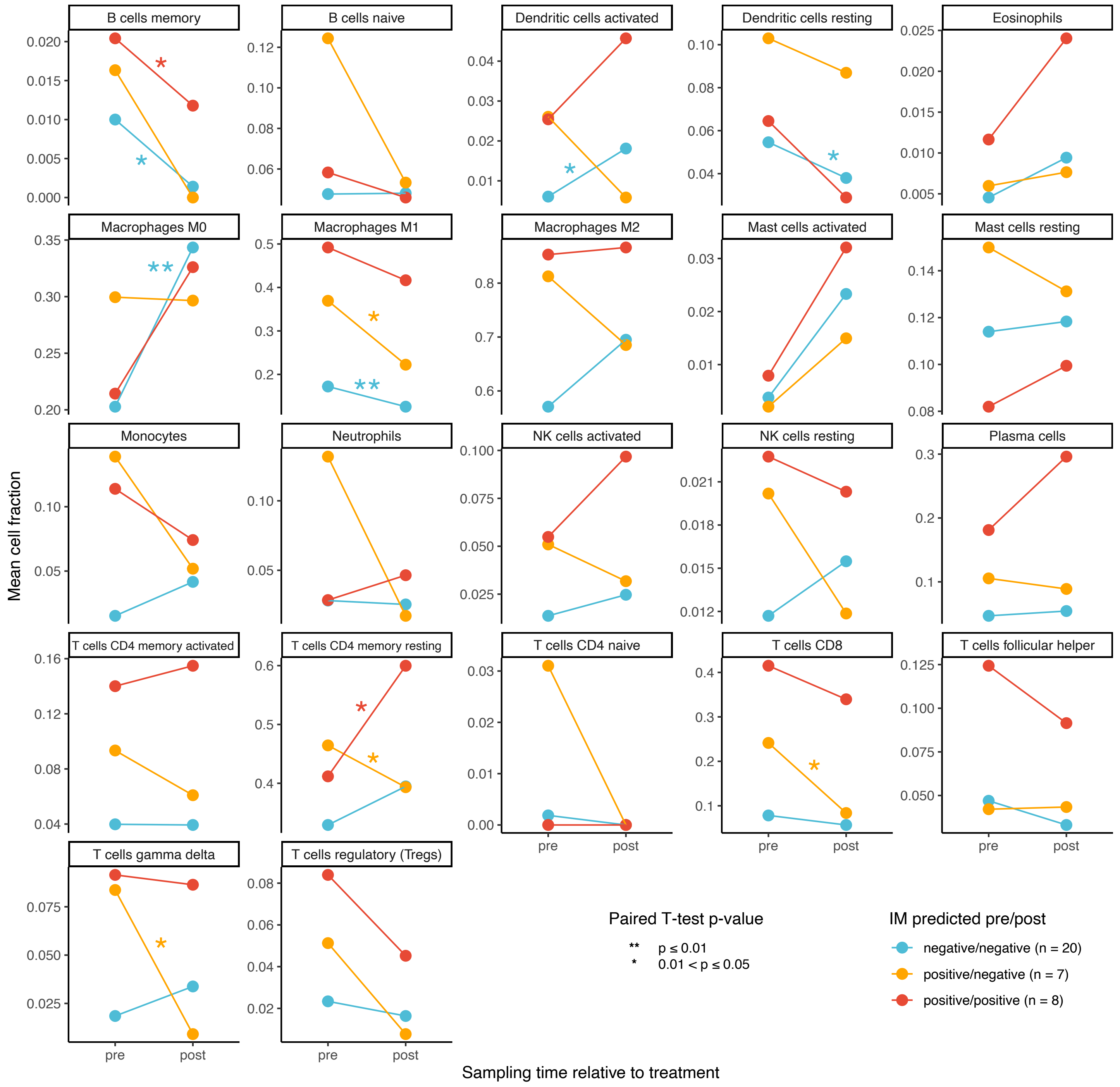

Supplementary Figure 6. Immune cell fraction estimates for samples obtained pre- and post-treatment. Mean cell fractions for 22 immune cell types estimated with a deconvolution approach (CIBERSORTx) from RNAseq data vs different groups considering IM predicted status pre- and post-treatment. Values for the one patient predicted to be IM-negative/positive are not shown. Asterisks refer to unadjusted p-values of two-sided paired T-tests. All comparisons not shown were not significant (p-value > 0.05).
