# Supplementary material for: Stand-alone Transcriptional Immune Response Prediction in Primary Triple-Negative Breast Cancer
Source: Cancer Res Commun. 2025 Dec 15;5(12):2157–74. doi: 10.1158/2767-9764.CRC-25-0453 (PMC12703016; doi:10.1158/2767-9764.CRC-25-0453)
Supplement: Supplementary Figure 7 — showing immune metagene rank scores for the IM classifier applied to 23 FUSCC_validation tumors with no IM consensus label from the online TNBCtype tool. [file crc-25-0453_supplementary_figure_7_suppsf7.pdf]

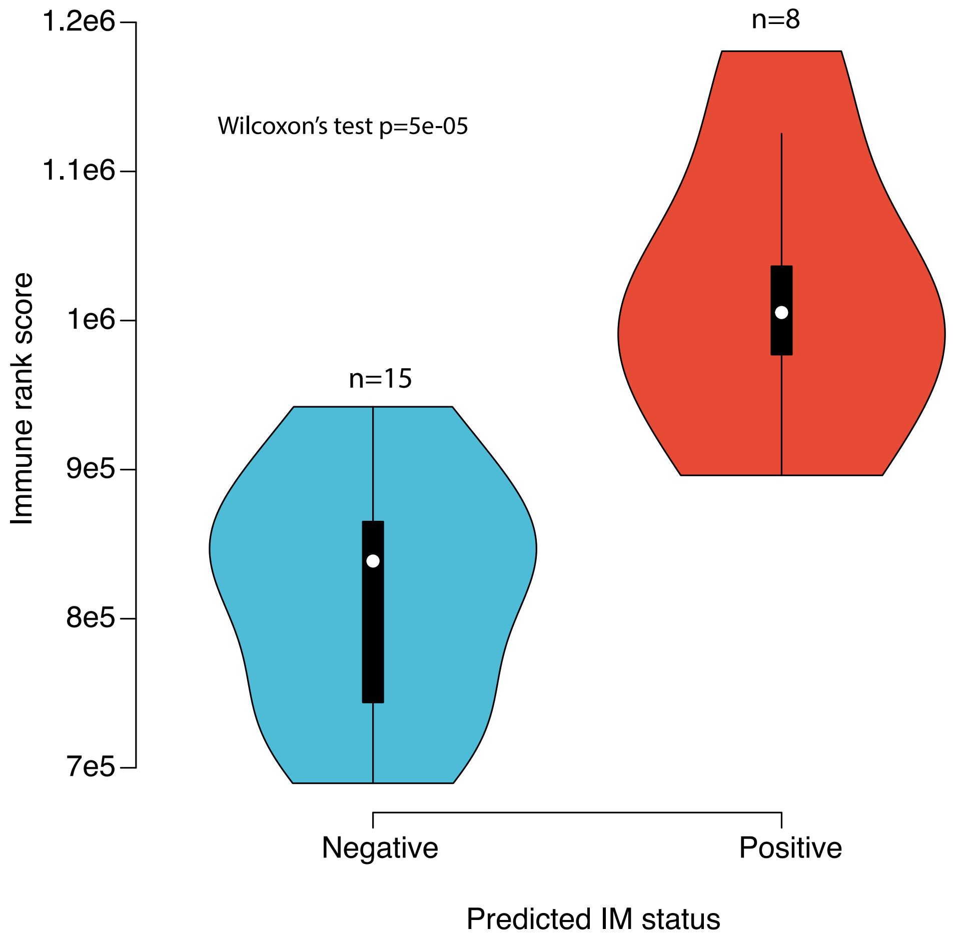

Supplementary Figure 7. Immune metagene rank scores for the IM classifier applied to 23 FUSCC_validation tumors with no IM consensus label from the online TNBCtype tool.
